# Dynamics of histone acetylation during human early embryogenesis

**DOI:** 10.1038/s41421-022-00514-y

**Published:** 2023-03-14

**Authors:** Keliang Wu, Dongdong Fan, Han Zhao, Zhenbo Liu, Zhenzhen Hou, Wenrong Tao, Guanling Yu, Shenli Yuan, Xiaoxiao Zhu, Mengyao Kang, Yong Tian, Zi-Jiang Chen, Jiang Liu, Lei Gao

**Affiliations:** 1 Center for Reproductive Medicine, National Research Center for Assisted Reproductive Technology and Reproductive Genetics, Key laboratory of Reproductive Endocrinology for Ministry of Education, Shandong University, Jinan, Shandong China; 2 University of Chinese Academy of Sciences, Beijing, China; 3 Key Laboratory of RNA Biology of CAS, Institute of Biophysics, Chinese Academy of Sciences, Beijing, China; 4 CAS Key Laboratory of Genome Sciences and Information, Beijing Institute of Genomics, Chinese Academy of Sciences and China National Center for Bioinformation, Beijing, China; 5 Shanghai Key Laboratory for Assisted Reproduction and Reproductive Genetics, Shanghai, China; 6 Shandong Key Laboratory of Reproductive Medicine, Shandong Provincial Hospital Affiliated to Shandong First Medical University, Jinan, Shandong China; 7 Center for Excellence in Animal Evolution and Genetics, Chinese Academy of Sciences, Kunming, Yunnan China

**Keywords:** Reprogramming, Histone analysis

## Abstract

It remains poorly understood about the regulation of gene and transposon transcription during human early embryogenesis. Here, we report that broad H3K27ac domains are genome-widely distributed in human 2-cell and 4-cell embryos and transit into typical peaks in the 8-cell embryos. The broad H3K27ac domains in early embryos before zygotic genome activation (ZGA) are also observed in mouse. It suggests that broad H3K27ac domains play conserved functions before ZGA in mammals. Intriguingly, a large portion of broad H3K27ac domains overlap with broad H3K4me3 domains. Further investigation reveals that histone deacetylases are required for the removal or transition of broad H3K27ac domains and ZGA. After ZGA, the number of typical H3K27ac peaks is dynamic, which is associated with the stage-specific gene expression. Furthermore, P300 is important for the establishment of H3K27ac peaks and the expression of associated genes in early embryos after ZGA. Our data also indicate that H3K27ac marks active transposons in early embryos. Interestingly, H3K27ac and H3K18ac signals rather than H3K9ac signals are enriched at ERVK elements in mouse embryos after ZGA. It suggests that different types of histone acetylations exert distinct roles in the activation of transposons. In summary, H3K27ac modification undergoes extensive reprogramming during early embryo development in mammals, which is associated with the expression of genes and transposons.

## Introduction

Mammalian embryo development begins from a fertilized zygote. After fertilization, most of genes are transcriptionally inactive. Major zygotic genome activation (ZGA) occurs at the late 2-cell stage in mouse, and at the 8-cell stage in human^1^. After ZGA, embryonic cells begin to have distinct cell identities, and they differentiate into inner cell mass (ICM) and trophectoderm (TE) at the blastocyst stage^2^. Mammalian embryos present genome-wide reprogramming of DNA methylation^3–6^, histone modification^7–11^, chromatin accessibility^12–14^ and 3D chromatin structure^15,16^. The epigenetic reprogramming is believed to function in the temporal regulation of gene expression. However, most of previous studies only show the correlation between epigenetic dynamics and gene expression. Recently, it is shown that the removal of broad H3K4me3 domains is required for normal ZGA^7^. Very limited is known about the regulatory mechanisms of the reprogramming of epigenetic information in gene expression in early embryos after the ZGA.

Histone modifications are involved in the regulation of gene expression during development^17^. They usually serve as indicators of chromatin states. For instance, H3K4me3 is enriched at the active promoters, while H3K27ac usually marks active enhancers^18^. The landscapes of histone modifications have been extensively investigated in human and mouse early embryos^7–11^. Non-canonical H3K4me3 broad domains are observed in both human and mouse embryos before ZGA, which are inherited from oocytes in mouse but are de novo established in human early embryos^7,11^. Blocking the removal of broad H3K4me3 domains can inhibit normal ZGA and embryonic development in mouse^7^. A recent study has also examined H3K27ac distribution in early embryos. Dahl et al. only found canonical H3K27ac peaks, but not broad domains, in mouse oocytes, 2-cell and 8-cell embryos^7^. The investigation of H3K27ac patterns in human embryos at the 8-cell and blastocyst stages shows that the stage-specific H3K27ac signal is associated with stage-specific gene expression, which is consistent with the key developmental events in human early embryo^11^. However, it remains unknown about the dynamics and the potential functions of H3K27ac around ZGA stage during human embryogenesis.

Besides genes, retrotransposons are also transcribed in human early embryos^19,20^. Because active retrotransposons can result in random insertions in the genome, in most circumstances, retrotransposons are repressed in mature cells. Previous studies mainly focused on the epigenetic information such as DNA methylation, PIWI-interacting RNA and H3K9me3 that serve as epigenetic repressors for transposon expression^21^. However, it remains unknown whether there is an epigenetic activator for transposon expression in early embryos.

To address these questions, we investigated the dynamics and functions of H3K27ac in human early embryos.

## Results

### Dynamics of histone H3K27ac modifications during human embryogenesis

**Fig. 1:**
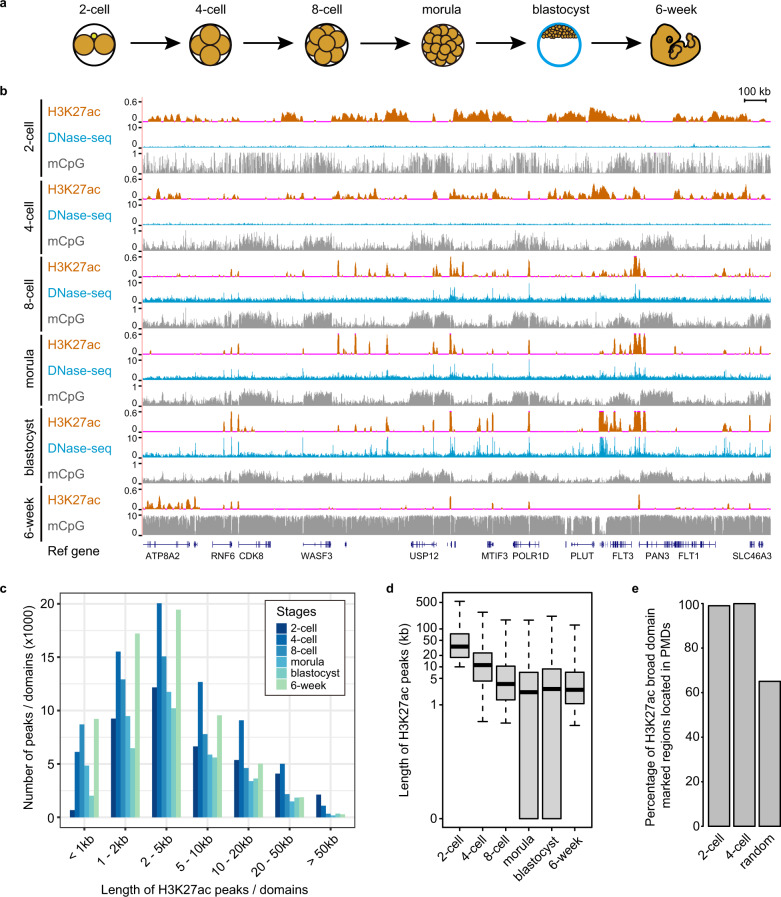
Dynamics of broad H3K27ac domains in human early embryos development. **a** Schematic diagram of human early embryos collected for H3K27ac ChIP-seq analysis. **b** Genome browser snapshot of H3K27ac enrichment, DNase-seq signal and DNA methylation levels in human early embryos. The published DNase-seq data^13^ and DNA methylome data^5^ are used. **c** The numbers of H3K27ac peaks or domains within different ranges of lengths in human early embryos. **d** Box plots showing the lengths of H3K27ac peaks located in the genomic regions with 2-cell broad H3K27ac domains (> 10 kb) in human embryos at different stages. Boxes and whiskers represent the 25th/75th percentiles and 1.5× interquartile range, respectively. **e** Percentage of broad H3K27ac domains located in PMDs at the 2-cell and 4-cell stages, respectively. The percentage of random regions located in the PMDs at the 2-cell stage is used for comparison.

In order to investigate the functions of H3K27ac landscapes in human early embryos, we developed a low-input ChIP-seq method with ~50 cells by optimizing recently published methods^22–24^. We validated this method by mapping H3K27ac modification in mouse 8-cell embryos. The ChIP-seq results are highly reproducible and consistent with the published data which were obtained from 500 cells (Supplementary Fig. S1a–c)^7^. A large percentage (80.3%) of peaks discovered by our method are also present in the published data (Supplementary Fig. S1d)^7^. The results suggest that our ChIP-seq method is robust. Using this method, we performed ChIP-seq assays for H3K27ac in human early embryos at the 2-cell, 4-cell, 8-cell, morula, blastocyst and 6-week stages (Fig. 1a; Supplementary Table S1). At least two replicates were performed for each stage (Supplementary Fig. S2a, b). We find a genome-wide distribution of broad H3K27ac domains which span > 10 kb long in human 2-cell and 4-cell embryos, many of which are even longer than 50 kb (Fig. 1b, c). In contrast, the broad H3K27ac domains that are longer than 50 kb are seldomly observed in embryos at the 8-cell stage and beyond (Fig. 1b, c). 16.3% and 17.4% of human genome show enriched H3K27ac signal at the 2-cell stage and 4-cell stage, respectively (Supplementary Fig. S2c). In particular, 13.1% of genome are covered by broad H3K27ac domains (> 10 kb) at the 2-cell stage (Supplementary Fig. S2c). However, less than 10% of human genome are covered by H3K27ac signal after ZGA (Supplementary Fig. S2c). It also indicates that the genomic regions marked by broad H3K27ac domains are narrowed down during development. At the 8-cell stage and onward, most of broad H3K27ac domains (> 10 kb) detected in 2-cell embryos disappear or transit to narrow H3K27ac peaks (Fig. 1b–d; Supplementary Fig. S2d), suggesting that the disappearance of broad H3K27ac domains is associated with ZGA in human.

To validate the observation of broad H3K27ac domains in human early embryos, we performed H3K27ac immunostaining with human embryos. The results show that the signal of H3K27ac modification is weak in the oocytes, and becomes strong in zygotes, 2-cell and 4-cell embryos, and then changes to be very weak in 8-cell embryos (Supplementary Fig. S2e). The results support our observation that much more broad H3K27ac domains are observed in the 2-cell and 4-cell embryos than the 8-cell embryos (Fig. 1c). The strong signal in the zygotes may suggest that broad H3K27ac domains also exist in the zygotes.

Next, we characterized the features of H3K27ac-enriched regions in human early embryos. Firstly, the majority of H3K27ac-enriched regions are located in the intergenic and intron regions (Supplementary Fig. S3a). Compared to the random regions, the H3K27ac-enriched regions are more enriched in promoters (Supplementary Fig. S3a). Secondly, we analyzed H3K27ac signal in the promoters of protein-coding genes. Our data show that H3K27ac signal is spread across promoters at the 2-cell and 4-cell stages (Supplementary Fig. S3b). The H3K27ac signal is depleted at the transcription start sites (TSSs) (Supplementary Fig. S3b). In total, 84.7% of promoters (16,300 in 19,235 promoters) show enriched H3K27ac signal at the 2-cell and 4-cell stages. Among them, 70.6% of the promoters (11,501/16,300) are covered by broad H3K27ac domains (> 10 kb). Comparing to the 2-cell and 4-cell embryos, H3K27ac signal around TSSs becomes concentrated around TSSs after the 8-cell stage (Supplementary Fig. S3b, c). For the promoters with significant enrichment of H3K27ac signal at the 8-cell stage, 96.5% of them (12,986 in 13,444 promoters) show enriched H3K27ac signal at the 2-cell or 4-cell stages, with 9461 promoters (72.9%, 9461/12,986) covered by broad H3K27ac domains (> 10 kb). It suggests that the majority of promoters with H3K27ac at ZGA stage are marked by broad H3K27ac domains before ZGA. Notably, the promoters covered by broad H3K27ac domains show higher CpG densities than those without broad H3K27ac signal (Supplementary Fig. S3b, d). In human early embryos, a large proportion of human genome are partially methylated domains (PMDs)^3–5^. We examined the relationship between the genomic regions covered by broad H3K27ac domains and PMDs. Our data show that > 95% of broad H3K27ac domains overlap with PMDs (Fig. 1e). Consistently, DNA methylation levels of broad H3K27ac domains are significantly lower than those of the random regions at both 2-cell and 4-cell stages (Supplementary Fig. S3e).

Taken together, in human early embryos, broad H3K27ac domains are widely distributed in promoters and PMDs. Those broad domains are removed or turn into typical H3K27ac narrow peaks after ZGA.

### Broad H3K27ac domains highly overlap with broad H3K4me3 domains

**Fig. 2:**
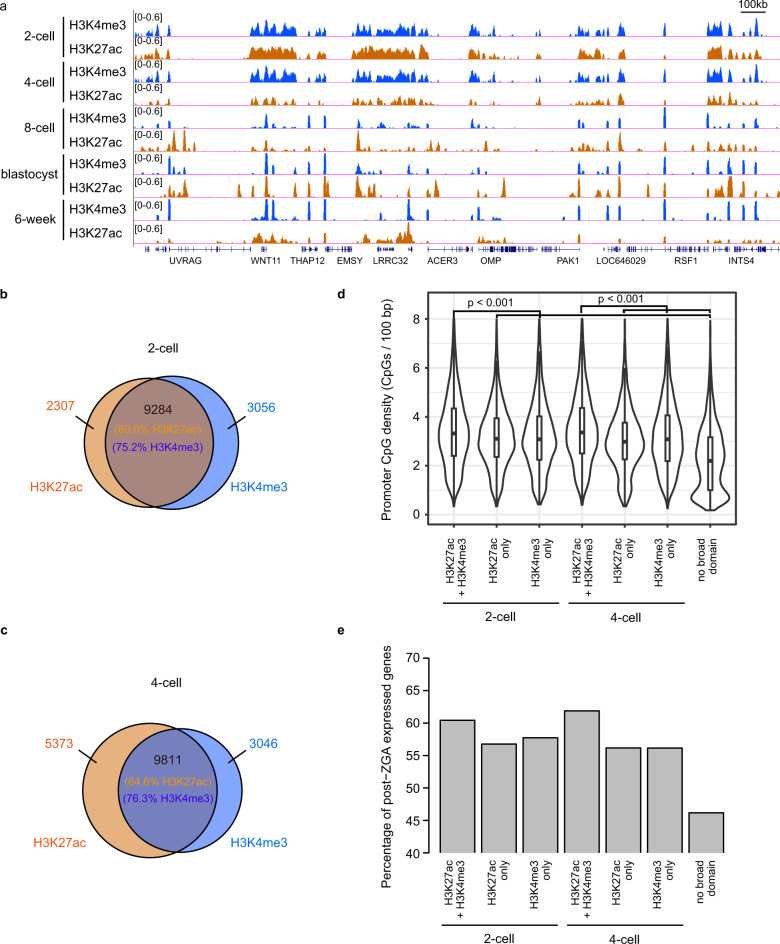
Broad H3K27ac domains almost show broad H3K4me3 signal patterns. **a** Genome browser snapshot of H3K27ac and H3K4me3 enrichment in human early embryos. **b** Venn diagram showing the number of overlapping peaks between broad H3K27ac domains and broad H3K4me3 domains in human 2-cell embryos. The percentages of overlapping broad domains are shown. **c** Venn diagram showing the number of overlapping peaks between broad H3K27ac domains and broad H3K4me3 domains in human 4-cell embryos. **d** Violin and box plots comparing CpG densities of promoters with broad H3K27ac or H3K4me3 domains (> 10 kb) in human 2-cell and 4-cell embryos. The promoters without broad H3K27ac or H3K4me3 domains are used for comparison. Wilcoxon rank sum test is used. Boxes and whiskers represent the 25th/75th percentiles and 1.5× interquartile range, respectively. **e** The percentages of genes expressed post-ZGA whose promoters are covered by broad H3K27ac or H3K4me3 domains (> 10 kb) before ZGA.

Previous work has described widespread H3K4me3 domains which are also enriched in PMDs in human 4-cell embryos^11^. We are curious about the relationship between broad H3K27ac domains and widespread H3K4me3 domains in human embryos before ZGA. Therefore, the H3K4me3 landscapes are also profiled in human early embryos from 2-cell stage to 6-week stage by using the low-input ChIP-seq method (Supplementary Fig. S4a). The H3K4me3 patterns in our data are highly correlated with the reported data^11^ (Supplementary Fig. S4b, Pearson’s correlation coefficients > 0.75). Consistently, widespread broad H3K4me3 domains (> 10 kb) are observed in human 2-cell and 4-cell embryos (Supplementary Fig. S4c). In particular, broad H3K4me3 domains which are > 50 kb can only be detected in human embryos at the 2-cell and 4-cell stages (Supplementary Fig. S4c). The H3K4me3-marked regions cover 14.7% and 13.0% of human genome at the 2-cell and 4-cell stages, respectively, while only < 9% of genome are covered by H3K4me3-marked regions at the other stages (Supplementary Fig. S4d). We further investigated the genomic distribution of H3K4me3 signal in human early embryos. The H3K4me3-marked regions are enriched in promoters at all stages comparing to the random regions (Supplementary Fig. S4e). Similar to H3K27ac signal, H3K4me3 signal in promoters becomes concentrated around TSS after ZGA stage (Fig. 2a; Supplementary Fig. S4f).

Next, we compared the distribution of broad H3K27ac and H3K4me3 domains (> 10 kb) in human 2-cell and 4-cell embryos. Our data show that about 75% of broad H3K4me3 domains overlap with broad H3K27ac domains at the 2-cell and 4-cell stages (Fig. 2a–c). It suggests that a large portion of human genome are covered by broad domains with both H3K27ac and H3K4me3 modifications in early embryos before ZGA stage. We further evaluated the genes associated with broad H3K27ac and H3K4me3 domains. Our results show that gene promoters with both broad H3K27ac and broad H3K4me3 signals harbor higher CpG densities than those with only broad H3K27ac domains or only broad H3K4me3 domains and those with neither broad domain (Fig. 2d). We also investigated the expression patterns of these genes in human early embryos. Compared to the genes whose promoters are not covered by broad H3K27ac or H3K4me3 domains, the genes whose promoters harbor both broad H3K27ac and H3K4me3 domains tend to be expressed at the post-ZGA stages (Fig. 2e; Supplementary Fig. S4g, Chi-square test *P*-values < 0.05).

### Broad H3K27ac domains are conserved between human and mouse

**Fig. 3:**
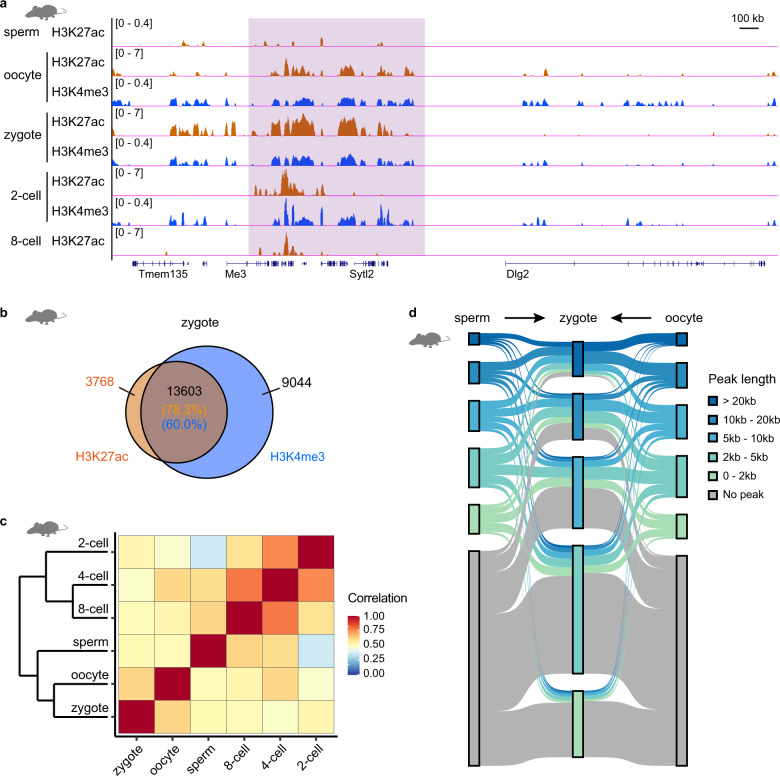
Broad H3K27ac domains also exist in mouse early embryos. **a** Genome browser snapshot of H3K27ac and H3K4me3 enrichment in mouse gametes and early embryos. The published H3K4me3 ChIP-seq data in mouse early embryos^7,9^ and H3K27ac ChIP-seq data in mouse sperm^61^ are used. Purple shadow marks a region with broad H3K27ac domains in mouse zygote. **b** Venn diagram showing the number of overlapping domains between broad H3K27ac domains (> 10 kb) and broad H3K4me3 domains (> 10 kb) in mouse zygote. The percentages of overlapping broad domains are shown. **c** Hierarchical clustering of H3K27ac patterns in mouse gametes and early embryos according to the Pearson’s correlation coefficients of H3K27ac signal between any two stages. **d** Sankey diagram showing the length dynamics of H3K27ac peaks from gametes to zygote in mouse. The dynamics of H3K27ac peaks in mouse zygote is shown.

Previous study did not examine H3K27ac modification in mouse embryos before the ZGA stage, and did not observe widespread broad H3K27ac domains in mouse early embryos^7^. Here, to answer whether the broad H3K27ac domains also exist in mouse early embryos before ZGA stage, we checked the H3K27ac modification in mouse PN5 zygotes, as well as 2-cell and 8-cell embryos. Our data show that many broad H3K27ac domains with lengths > 10 kb, especially those with lengths > 20 kb, are widely detected in mouse zygotes (Fig. 3a; Supplementary Fig. S5a). In contrast, broad H3K27ac domains (> 20 kb) are seldomly observed in mouse embryos after ZGA (Fig. 3a; Supplementary Fig. S5a). 17.6% of mouse genome are covered by H3K27ac signals in mouse zygotes, while < 10% of genome are covered by H3K27ac signals in mouse embryos after ZGA (Supplementary Fig. S5b), which is consistent with previous report^7^. Furthermore, immunostaining results show that H3K27ac signal in mouse zygote is stronger than that in mouse 2-cell embryo (Supplementary Fig. S5c), which supports the existence of widespread broad H3K27ac domains in mouse zygote. In addition, the broad H3K27ac domains detected in the embryos before ZGA is not derived from technical artifact (Supplementary Fig. S5d–f). Next, we asked whether broad H3K27ac domains also largely overlapped with broad H3K4me3 domains in mouse embryos before ZGA stage as observed in human. Our data show that about 78.3% of broad H3K27ac domains are located in broad H3K4me3 domains in mouse zygote (Fig. 3b).

Previous studies have revealed that broad H3K4me3 domains in mouse early embryo before ZGA are inherited from oocyte^7,9^. To answer whether broad H3K27ac domains in mouse zygote are inherited from oocyte or sperm, we profiled H3K27ac pattern in mouse MII oocyte, and compared the H3K27ac patterns in mouse gametes and zygote (Fig. 3a; Supplementary Fig. S6a). Our data show that the genome-wide distribution of H3K27ac signal in mouse zygote is much similar to that in mouse oocyte as compared with those in sperm and early embryos after ZGA (Fig. 3a, c). We also noticed that much more broad H3K27ac domains with length > 20 kb could be detected in zygote than oocyte and sperm (Supplementary Fig. S5a). It suggests that the H3K27ac pattern is reprogrammed after fertilization. We further compared the H3K27ac-enriched regions among mouse gametes and zygote (Fig. 3d). The results show that more H3K27ac-enriched regions are inherited from oocyte than sperm. For narrow H3K27ac peaks with length < 10 kb in mouse zygote, 68.3% of them are de novo established in the regions without H3K27ac enrichment in mouse gametes (Fig. 3d). For broad H3K27ac domains with length > 10 kb, 8.0% of them are inherited only from oocyte, 4.8% of them are inherited only from sperm, and 0.5% of them are inherited from the shared broad domains between oocyte and sperm (Fig. 3d; Supplementary Fig. S6b). The majority of broad H3K27ac domains in mouse zygote are de novo established through extensive spreading of H3K27ac peaks in gametes after fertilization (Fig. 3d). We also compared the H3K27ac patterns between sperm and 2-cell embryo in human (Supplementary Fig. S6c). Similar to the observation in mouse, only 0.4% of broad H3K27ac domains in human 2-cell embryo are inherited from sperm (Supplementary Fig. S6d). The majority of broad H3K27ac domains in human 2-cell embryo are not inherited from sperm.

Taken together, broad H3K27ac domains are widely distributed in the embryos before the ZGA in both human and mouse.

### Histone deacetylases (HDACs) are required for the transition of broad H3K27ac domains to narrow peaks and ZGA

**Fig. 4:**
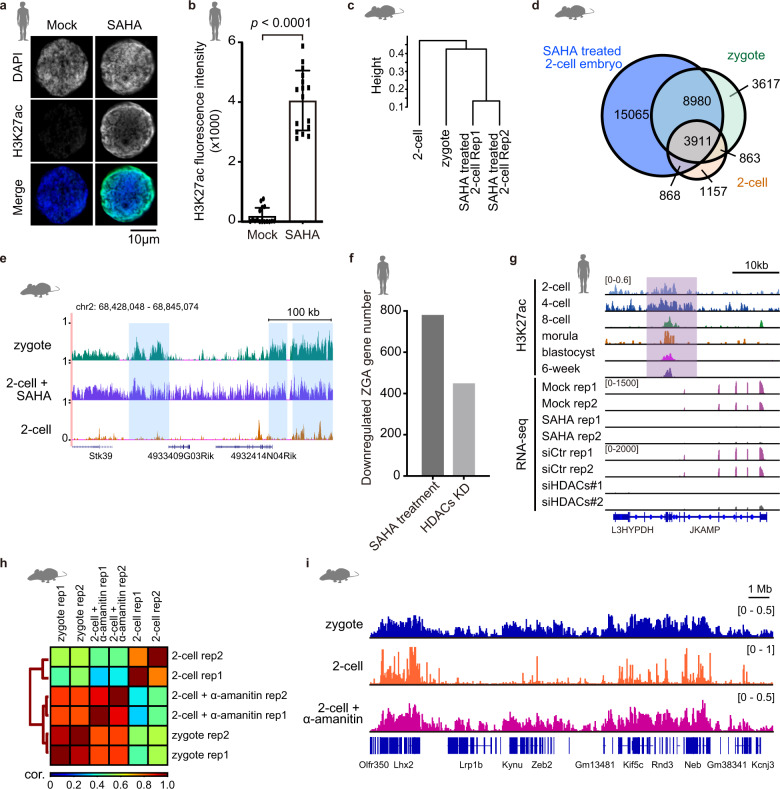
HDACs are essential for the removal or transition of broad H3K27ac domains and ZGA in human. **a** Immunostaining of H3K27ac for human 8-cell embryos with or without SAHA treatment. The representative images of 2 independent experiments are shown. Each panel indicates the DAPI or H3K27ac signal in a nucleus of an 8-cell embryo. **b** Quantification of the H3K27ac fluorescence intensities in human 8-cell embryos with mock or SAHA treatment. 16 cells of two 8-cell embryos were quantified for each group. Error bars represent standard errors. *t*-test is used. **c** Hierarchical clustering of H3K27ac patterns in mouse zygotes, 2-cell embryos and SAHA-treated 2-cell embryos. **d** Venn diagram showing the numbers of overlapping broad H3K27ac domains (> 10 kb) among zygotes, 2-cell embryos and SAHA-treated 2-cell embryos in mouse. **e** Genome browser view of H3K27ac enrichment in mouse zygotes, 2-cell embryos and SAHA-treated 2-cell embryos. Blue shadows mark broad H3K27ac domains in mouse zygotes. **f** The number of downregulated human ZGA genes in SAHA-treated or HDACs KD 8-cell embryos. HDACs KD represents HDAC1, HDAC2 and HDAC3 triple knockdown by siRNAs. **g** Genome browser view of H3K27ac enrichment at *JKAMP* gene locus in human early embryos, and RNA-seq signals of human *JKAMP* gene in control, SAHA-treated or HDAC KD 8-cell embryos. **h** Hierarchical clustering of H3K27ac patterns in mouse zygotes, 2-cell embryos and α-amanitin-treated 2-cell embryos according to the Pearson’s correlation coefficients of H3K27ac signal between any two samples. **i** Genome browser view of H3K27ac enrichment in mouse zygotes, 2-cell embryos and α-amanitin-treated 2-cell embryos.

In mammals, HDACs can remove the acetylation from H3K27ac^25,26^. We are curious about whether HDACs are required for the transition of H3K27ac modifications from broad domains to narrow peaks during human embryo development. We firstly investigated the gene expression of HDACs in human early embryos. The results show that human HDAC1, HDAC2 and HDAC3 are highly expressed at the 2-cell, 4-cell and 8-cell stages (Supplementary Fig. S7a). The activities of these three HDACs can be blocked by suberoylanilide hydroxamic acid (SAHA)^27^. To check whether HDACs mediate the deacetylation of H3K27ac in broad domains, we treated the human zygotes with SAHA. Immunostaining images show that the level of H3K27ac modifications in SAHA-treated 8-cell embryos is extraordinarily higher than that in control 8-cell embryos, suggesting that the deacetylation of H3K27ac during human ZGA is mediated by HDACs (Fig. 4a, b). It is conserved that Hdac1, Hdac2 and Hdac3 are also expressed in mouse zygote and 2-cell embryo, and H3K27ac signal is much stronger in SAHA-treated mouse 2-cell embryos comparing to the control embryos (Supplementary Fig. S7b, c). To further confirm that the transition of broad H3K27ac domains was disrupted upon the inhibition of HDACs, we checked the H3K27ac signal in mouse late 2-cell embryos which were treated with SAHA from zygote stage. The H3K27ac pattern in 2-cell embryo upon SAHA treatment resembles that in zygote rather than 2-cell embryo (Fig. 4c). Broad H3K27ac domains are widely distributed in SAHA-treated mouse late 2-cell embryos. 74.2% (12,891/17,371) of broad H3K27ac domains (> 10 kb) in mouse zygote can still be detected in the SAHA-treated mouse 2-cell embryos (Fig. 4d, e). Furthermore, 8980 of broad H3K27ac domains in zygote that were disappeared at the 2-cell stage can still be observed in the SAHA-treated 2-cell embryos (Fig. 4d, e). Taken together, HDACs are required for the transition of broad H3K27ac domains to narrow peaks at the ZGA stage.

We further asked whether HDACs inhibition also disrupted human ZGA. To know how many genes are activated in human ZGA, we compared the transcriptomes of 2-cell and 8-cell human embryos, and then defined 2863 genes as ZGA genes which were significantly upregulated in 8-cell embryos (fold change > 3)^13^. To examine the influence of HDACs inhibition on the expression of ZGA genes, we measured the gene expression of human 8-cell embryos treated with SAHA (Supplementary Table S2). Gene expression data show that 27.1% (777/2863) of ZGA genes are downregulated in SAHA-treated 8-cell embryos (Fig. 4f). Notably, 97.8% (760/777) of the downregulated ZGA genes are covered by broad H3K27ac domains around TSS at the 4-cell stage (Fig. 4g). To further confirm the function of HDACs on human ZGA, we knocked down HDAC1, HDAC2 and HDAC3 simultaneously in human zygotes (Supplementary Table S2). RNA expression levels were analyzed at the 8-cell stage (Supplementary Fig. S7d). Our data show that 446 ZGA genes are downregulated in the knockdown (KD) 8-cell embryos (Fig. 4f). Among them, 209 ZGA genes are also downregulated in SAHA-treated 8-cell embryos. RNA expression profiles of HDACs KD 8-cell embryos resemble those of SAHA-treated embryos (Fig. 4g; Supplementary Fig. S7e). Taken together, these results suggest that HDACs are required for human ZGA. It also indicates that transition of broad H3K27ac domains to typical peaks catalyzed by HDACs is associated with the ZGA in human.

The majority of H3K27ac broad domains are removed after the ZGA. However, a limited proportion of human genome are still covered by H3K27ac broad domains from the 8-cell stage onward (Fig. 1c; Supplementary Fig. S7f). The genes nearby H3K27ac broad domains show higher expression level than those nearby typical narrow H3K27ac peaks (Supplementary Fig. S7g). These H3K27ac domains are previously defined as super-enhancers^28^. H3K27ac broad domains from the 8-cell onward are positively correlated with gene expression.

Since the transition of broad H3K27ac domains into typical peaks during ZGA is observed in both human and mouse early embryos, it suggests that the underlying mechanism regulating this process is conserved between human and mouse. To answer whether the removal of broad H3K27ac domains is ZGA-dependent, we treated mouse zygotes with alpha-amanitin, which could inhibit RNA transcription^9,29,30^. The alpha-amanitin-treated late 2-cell embryos were collected for H3K27ac ChIP-seq analysis. Our data show that the H3K27ac landscape in alpha-amanitin-treated late 2-cell embryos is similar to that in zygote rather than that in control late 2-cell embryos (Fig. 4h). Moreover, compared to the typical H3K27ac peaks observed in mouse late 2-cell embryos, broad H3K27ac domains can be widely detected in the alpha-amanitin-treated late 2-cell embryos (Fig. 4i). These results suggest that the removal of broad H3K27ac domains is ZGA-dependent. Consistently, HDACs, especially HDAC1, are highly expressed at the ZGA stages in both human and mouse (Supplementary Fig. S7a, b).

### The dynamics of typical H3K27ac peaks during early embryogenesis

**Fig. 5:**
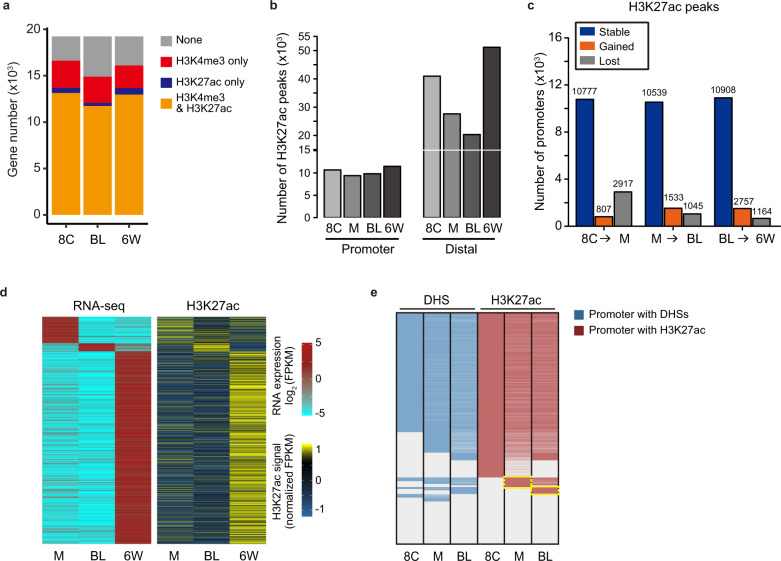
Dynamics of canonical H3K27ac peaks after ZGA in human early embryo. **a** Number of H3K27ac- and H3K4me3-marked promoters in human embryos. 8C, 8-cell embryo; BL, blastocyst; 6 W, 6-week embryo. **b** Bar plots showing the numbers of promoter H3K27ac peaks or distal H3K27ac peaks in human early embryos. 8C, 8-cell embryo; M, morula; BL, blastocyst; 6 W, 6-week embryo. **c** Plot showing the dynamics of promoters with H3K27ac enrichment between adjacent two stages. Promoter number is indicated above each bar. **d** Heat map showing the RNA expression and H3K27ac signal in the promoters of stage-specifically expressed genes (*n* = 3225). **e** Heat map showing DHSs and H3K27ac enrichment in the promoters of all protein-coding genes at 8-cell, morula and blastocyst stages. The dashed line rectangles label the promoters with H3K27ac signals gained at the morula and blastocyst stages.

At the 8-cell stage, the broad H3K27ac domains transit into typical H3K27ac peaks in human (Fig. 1b). We find that 77.7% of H3K27ac peaks are located in the distal regions (non-promoter regions) at the 8-cell stage (Supplementary Fig. S3a). About 71.1% (13,694 in 19,235) of human promoters show H3K27ac enrichment at the 8-cell stage. Expression analysis show that genes with promoter H3K27ac enrichment are highly expressed (Supplementary Fig. S8a). We also noticed that most of H3K27ac-marked promoters also harbored H3K4me3 modification (Fig. 5a), and promoters with both modifications corresponded to higher levels of gene expression (Supplementary Fig. S8b). During human early embryo development from the 8-cell stage to 6-week stage, H3K27ac peaks in promoter regions are more stable than distal H3K27ac peaks (Fig. 5b, c). Similarly, the numbers of H3K4me3-marked promoters rarely change (Supplementary Fig. S8c). In spite of this, many genes are stage-specifically expressed in human morula, blastocyst and 6-week embryos. Notably, the expression of stage-specific genes corresponds to stage-specific H3K27ac modifications in promoters (Fig. 5d). These data suggest that the establishment of H3K27ac modification in promoters plays important role in temporal regulation of gene expression during human embryogenesis. It has been shown that the number of promoters with DNase I hypersensitive site (DHSs) is significantly increased after the 8-cell stage during human development^13^. Our data show that the majority of promoters with DHSs in human early embryos show H3K27ac enrichment as early as 8-cell stage. (Fig. 5e). In addition, we find that some promoters establishing H3K27ac at the morula and blastocyst stages have already established DHSs at the 8-cell stage (Fig. 5e, yellow box). It suggests that the stage of establishing H3K27ac for some promoters is later than that of establishing DHSs.

### Putative enhancers in human early embryos

Distal H3K27ac peak is reported to be a marker of putative active enhancer^31^. To investigate the putative enhancers in human early embryos, we used stringent criteria to call narrow peaks with H3K27ac enrichment (see Methods). Consistently, genes with nearby distal H3K27ac peaks show higher expression levels than those without (Supplementary Fig. S9a). DHS is another indicator of *cis*-regulatory element. We find that 80.8% (16,353 in 20,239) of distal H3K27ac peaks have nearby DHS sites (within 500 bp) in blastocysts (Supplementary Fig. S9b, c). We called the regions marked by both distal H3K27ac modifications and nearly DHSs as putative enhancers. Among these putative enhancers in human blastocysts, 77 of them are experimentally validated human enhancers in VISTA database^32^ (Supplementary Fig. S9d).

Next, we performed a stage-specific analysis of the putative enhancers^33^ (Supplementary Fig. S9e). A total of 7267 stage-specific enhancers are discovered at the 8-cell, morula, blastocyst and 6-week stages. Gene expression analysis show that stage-specific enhancer-associated genes (with the closest TSSs around putative enhancers) show high expression levels at the corresponding stages in early embryos (Supplementary Fig. S9f). The associated genes of 8-cell-specific enhancers are enriched in viral process, chromosome organization and RNA processing; the associated genes of blastocyst-specific enhancers are enriched in cytoskeletal anchoring at plasma membrane and endothelial cell development; and the associated genes of 6-week-specific enhancers are enriched in skeletal system development and circulatory system development (Supplementary Fig. S9g). These results suggest that stage-specific enhancers are associated with the occurrence of key events at the corresponding stages.

Then we performed the analyses of transcriptional factor binding motif enrichments in these putative active enhancers. We demonstrate that GSC- and OTX2-binding motifs are enriched in 8-cell-specific putative active enhancers, OCT4- and KLF5-binding motifs are enriched in morula-specific putative active enhancers, GRHL2- and TEAD1-binding motifs are enriched in blastocyst specific putative enhancers, and PITX1- and TCF21-binding motifs are enriched in 6-week embryo putative enhancers (Supplementary Fig. S10a, b). PITX1 plays important roles in the development of the brain, face and hind limb, which is consistent with the development of fetal brain and limb buds taking place in the 6-week embryos^34–36^. Gene expression data also show that those transcription factors are expressed at the corresponding stages (Supplementary Fig. S10b). Taken together, the dynamics of H3K27ac is associated with the process of embryogenesis in human.

### P300 is essential for the establishment of canonical H3K27ac modifications and gene expression after ZGA

**Fig. 6:**
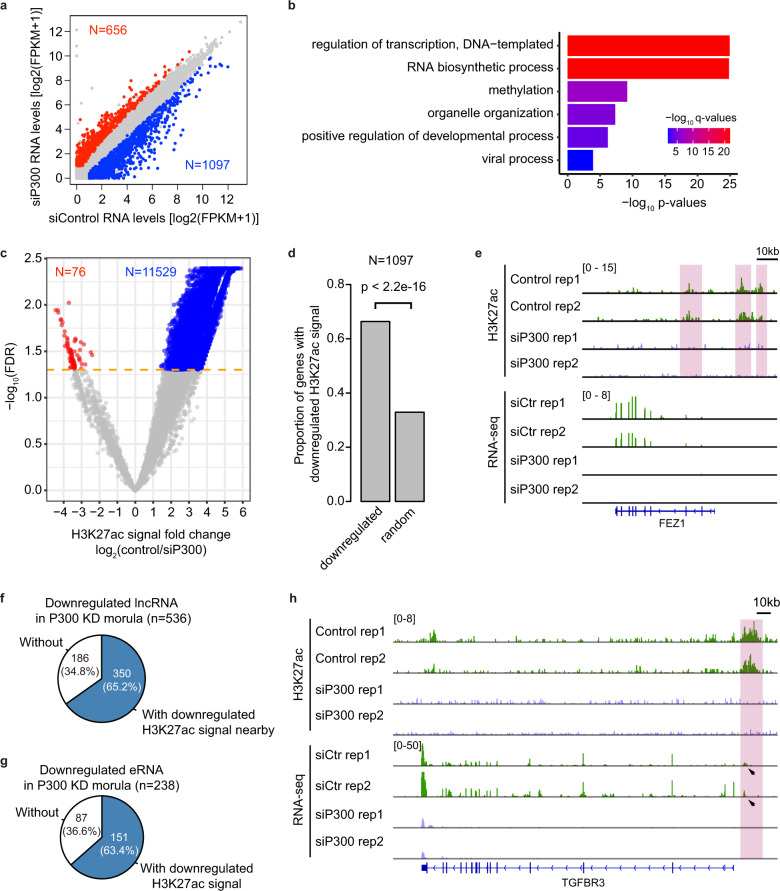
P300 is essential for the establishment of H3K27ac modification and gene expression in human early embryos. **a** Scatter plot showing the RNA expression levels in control KD embryos and P300 KD embryos at the morula stage. The blue dots represent the downregulated genes in P300 KD morula with fold changes > 2 and FDR values < 0.05 comparing to control KD embryos. The red dots represent the upregulated genes in P300 KD morula. The numbers of downregulated or upregulated genes in P300 KD morula are shown. Three biological replicates were performed for each group. **b** GO enrichment of the downregulated genes in P300 KD morula. Fisher exact test was used. The *P*-values were adjusted by Benjamini and Hochberg method. **c** Plot showing H3K27ac signal fold changes and FDR values of H3K27ac peaks in P300 KD embryos comparing to control embryos. The blue dots represent the H3K27ac peaks with downregulated H3K27ac signal in P300 KD morula with fold changes > 2 and FDR values < 0.05 comparing to control embryos. The red dots represent the H3K27ac peaks with upregulated H3K27ac signal in P300 KD morula. The numbers of peaks with downregulated or upregulated H3K27ac signal in P300 KD morula are shown. Two biological replicates were performed for each group. **d** Bar plot showing the proportion of genes with downregulated H3K27ac signal in P300 KD morula among the genes downregulated in P300 KD morula. The gene set including 1097 genes randomly chosen was used for comparison. Two-proportions z-test was used for statistical analysis. **e** Genome browser view of H3K27ac and RNA-seq patterns at *FEZ1* gene locus in control and P300 KD morulae. *FEZ1* shows downregulated H3K27ac signal and gene expression in P300 KD morula. Pink shadows highlight the H3K27ac peaks whose H3K27ac signals are depleted in P300 KD morula. **f** Pie chart showing the number and percentage of the lncRNA genes with or without downregulated H3K27ac signal in P300 KD morula among the lncRNA genes downregulated in P300 KD morula. **g** Pie chart showing the number and percentage of the eRNA with or without downregulated H3K27ac signal in P300 KD morula among the eRNA downregulated in P300 KD morula. **h** Genome browser view of H3K27ac and RNA-seq patterns of an eRNA around *TGFBR3* gene locus in control and P300 KD morulae. Pink shadow highlights the eRNA whose H3K27ac signal is depleted in P300 KD morula. The expression levels of the eRNA in control embryos are indicated by black arrows.

Until now, very limited is known about how gene expression is regulated by epigenetic information in early embryos after the ZGA. P300 is a histone acetylation transferase, which catalyzes histone H3K27 acetylation^37^. P300 is highly expressed in human early embryos (Supplementary Fig. S11a). To explore the role of H3K27ac on gene expression after the ZGA, we knocked down P300 in human zygote (Supplementary Fig. S11b, c and Table S2). At the morula stage, 1097 protein-coding genes are downregulated in P300 KD embryos (Fig. 6a). 78.1% (857 in 1097) of the downregulated genes show H3K27ac modifications nearby (within 50 kb around TSS) (Supplementary Fig. S11d). Moreover, 85.8% (941 in 1097) of those downregulated genes show P300 binding sites nearby (within 50 kb around TSS) in hESCs (Supplementary Fig. S11e, f). GO enrichment analysis reveals that the downregulated genes in P300 KD morula are enriched in the category of transcriptional regulation, methylation and viral process (Fig. 6b). To further confirm whether the gene downregulation in P300 KD morula is associated with the disruption of H3K27ac signal, we evaluated the H3K27ac pattern in P300 KD morulae. Our data show that the H3K27ac signals of 11,529 peaks are significantly downregulated (fold change > 2, false discovery rates (FDR) < 0.05) (Fig. 6c). Notably, for 1097 downregulated genes in p300 KD morulae, 66.3% of the genes (*n* = 728) show downregulated H3K27ac signals nearby (within 50 kb around TSS) (Fig. 6d, e). Besides, the genes with top 50% of H3K27ac signal fold changes in P300 KD morulae show larger decrease of RNA expression than the other downregulated genes upon P300 KD (Supplementary Fig. S11g). Taken together, our results suggest that P300 is important for the establishment of H3K27ac modifications and gene expression after the ZGA stage.

Next, we wanted to investigate the relationship between H3K27ac and non-coding RNA expression. We firstly focused on long non-coding RNA (lncRNA) (Supplementary Fig. S12a). As reported^38^, lncRNAs tend to be stage-specifically expressed in human early embryos (Supplementary Fig. S12b, c). Our data show that the expression levels of lncRNAs with H3K27ac are higher than those without H3K27ac (Supplementary Fig. S12d). Furthermore, the expression levels of lncRNAs are decreased in human morula upon P300 KD (Supplementary Fig. S12e). In particular, 536 of 16,171 lncRNA genes are significantly downregulated (Fig. 6f, fold change > 2, FDR < 0.05). Among the downregulated lncRNA genes, 65.2% (350/536) of the genes show decreased H3K27ac signal nearby in P300 KD morula comparing to control morula (Fig. 6f; Supplementary Fig. S12f). These results suggest that the transcription of 3% of lncRNAs depends on P300. H3K27ac signal is associated with the expression of many lncRNAs in human early embryos.

Enhancer RNA (eRNA) is another kind of non-coding RNA transcribed from enhancer regions^39^. Our data show that about 1500 eRNAs are expressed at each stage in human early embryos (Supplementary Fig. S12g). Consistent with the function of eRNA in enhancing gene expression, the eRNA enhancer-associated genes show higher expression levels than the non-eRNA enhancer-associated genes (Supplementary Fig. S12h). To answer whether the transcription of eRNA is dependent on the H3K27ac signal, we evaluated eRNA expression in P300 KD morula. Our data show that the genome-wide eRNA expression levels are significantly reduced in P300 KD morula compared to the control morula (Supplementary Fig. S12i). Specifically, for 1583 eRNAs that are expressed in human morula, 238 eRNAs are significantly downregulated in P300 KD morula. Among them, 63.4% (151/238) of eRNAs show disruption of H3K27ac signal upon P300 KD, which is associated with downregulation of 28 protein-coding genes (Fig. 6g, h). It suggests that P300 mediates the establishment of H3K27ac signal in many eRNAs, which is important for the expression of the eRNAs and associated genes.

Taken together, P300 is critical for the establishment of H3K27ac and the expression of H3K27ac-associated protein-coding and non-coding genes in human early embryos after ZGA stage.

### H3K27ac marks active transposons in early embryos

**Fig. 7:**
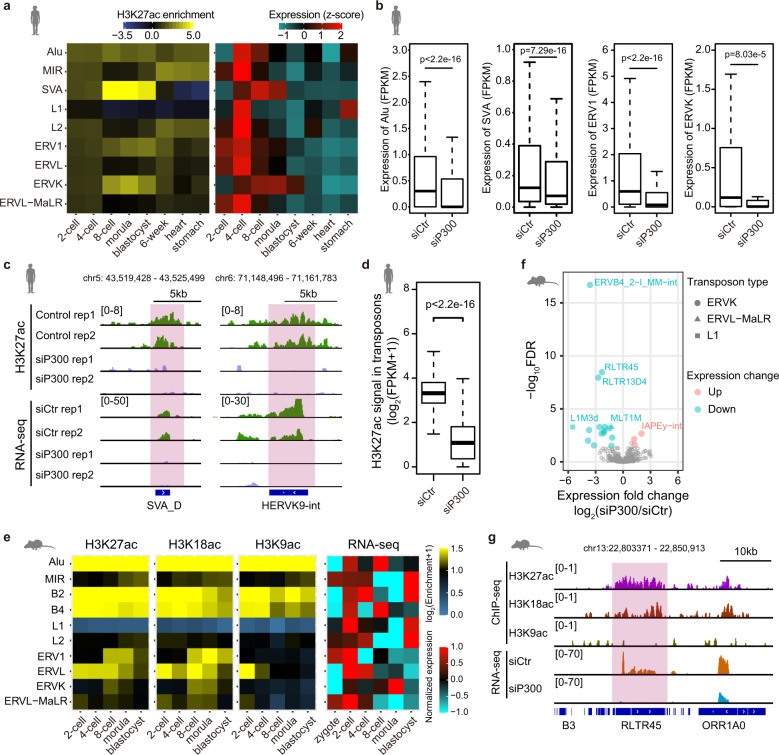
H3K27ac marks active retrotransposons. **a** Heat maps showing H3K27ac enrichments in retrotransposons and the expression of retrotransposons in human early embryos and primary tissues. The RNA-seq reads counts per million reads were used to represent the expression of retrotransposons. The expression values were scaled by z-scores. **b** Boxplots showing expression levels (FPKM values) of retrotransposons in control and P300 KD morulae. The expressed retrotransposons (FPKM > 0) with H3K27ac modifications in normal morula were used for comparison. Wilcoxon rank sum test is used. Boxes and whiskers represent the 25th/75th percentiles and 1.5× interquartile range, respectively. **c** Genome browser view of H3K27ac signal and RNA-seq signal of retrotransposons in control and P300 KD morula. Pink shadows indicate the genomic regions of SVA_D and HERVK9. **d** Boxplots comparing H3K27ac signals in transposons between control and P300 KD morulae. Wilcoxon rank sum test is used. Boxes and whiskers represent the 25th/75th percentiles and 1.5× interquartile range, respectively. **e** Heat maps showing H3K27ac, H3K18ac and H3K9ac enrichments in retrotransposons and the expression of retrotransposons in mouse early embryos. The RNA-seq reads counts per million reads were used to represent the expression of retrotransposons. The expression values were scaled by z-scores. **f** Plot showing expression fold changes and FDR values of transposons in mouse P300 KD 8-cell embryos comparing to control embryos. Only the transposons with expression fold changes > 2 and FDR values < 0.05 in P300 KD mouse 8-cell embryos are labeled by pink or steel blue colors. These transposons are further classified into different families. **g** Genome browser view of histone acetylation signals in mouse 8-cell embryo and RNA expression patterns in control or P300 KD mouse 8-cell embryos at an ERVK element. RLTR45 is an ERVK element. Pink shadow represents the genomic location of RLTR45.

Previous studies have shown transposons, especially human active retrotransposons, are highly expressed in early embryos^13,20^. In P300 KD human morula, the genes involved in viral process are downregulated, which implies that H3K27ac modification may play important role in the regulation of retrotransposons (Fig. 6b). Thus, we examined the relationship between H3K27ac and transposon expression. We find that H3K27ac modifications are highly enriched in SVA, ERVK, ERV1 and Alu at the 8-cell, morula and blastocyst stages (Fig. 7a). Importantly, these transposons are also highly expressed at the corresponding stages (Fig. 7a). We further compared the expression between transposons with and without H3K27ac, and showed that the expression levels of transposons with H3K27ac were higher than those without H3K27ac (Supplementary Fig. S13a–d). It suggests that the establishment of H3K27ac signal at transposons is associated with transposon activation. To further examine whether H3K27ac modification was important for the expression of these transposons, we checked the expression level of transposons in morula after P300 KD. Our data show that the expression of SVA, ERVK, ERV1 and Alu transposons with H3K27ac peaks are significantly reduced in P300 KD morula compared to the control morula (Fig. 7b, c). Notably, the H3K27ac signal in retrotransposons is significantly downregulated in P300 KD morula (Fig. 7c, d; Supplementary Fig. S13e–h). These data suggest that P300 is important for the establishment of H3K27ac signal in transposons and the transcription of the transposons. It also indicates that H3K27ac is associated with transposon expression.

Previous works have reported that P300 can catalyze the acetylation of multiple sites at the tail of histones, such as H3K27, H3K18 and H3K9^40^. To further illustrate whether the other types of histone acetylations also participated in the activation of transposons, we investigated the H3K27ac, H3K18ac and H3K9ac patterns in mouse early embryos, in which the retrotransposons were highly transcribed^41^ (Supplementary Tables S3 and S4). As conserved, H3K27ac signal is enriched in transposons in mouse (Fig. 7e). The enrichment of H3K18ac and H3K9ac in transposons can also be observed (Fig. 7e). Consistently, the transposons with histone acetylations are actively transcribed in mouse early embryos (Fig. 7e). Intriguingly, ERVK elements show high enrichment of H3K27ac and H3K18ac rather than H3K9ac at the 8-cell and morula stages (Fig. 7e). It suggests that different types of histone acetylations may play distinct roles in transposon activation in early embryos. In particular, H3K27ac and H3K18ac play more important roles in the activation of ERVKs than H3K9ac. To further validate the function of H3K27ac and H3K18ac in the activation of ERVKs in mouse early embryos, we knocked down P300 in mouse zygote, then checked the expression levels of transposons in P300 KD 8-cell embryos (Supplementary Fig. S13i). Our data indicate that ERVK elements are significantly downregulated in P300 KD 8-cell embryos (Fig. 7f, g).

Taken together, the establishment of H3K27ac modifications is associated with the activation of transposons in early embryos.

## Discussion

How gene expression is regulated during development is a fundamental question in biology. Our data show that the removal of broad H3K27ac domains is associated with normal ZGA in human. Previous studies show the removal of broad H3K4me3 domains is critical for normal ZGA in mouse^7^. Therefore, it seems that the establishment of both H3K4me3 and H3K27ac broad domains is a mechanism in silencing gene expression before the ZGA in mammals. Furthermore, we demonstrate that the establishment of canonical H3K27ac peaks is associated with gene expression at the morula stage (Fig. 6; Supplementary Fig. S13j). Therefore, the reprogramming of histone modifications may exert important functions in the temporal regulation of gene expression during embryogenesis.

Previous studies only find that non-canonical broad domains of H3K4me3, but not H3K27ac, are widely distributed in mouse early embryos. Here, we find that not only broad H3K4me3 domains but also H3K27ac domains are widely distributed in both human and mouse early embryos (Supplementary Fig. S13j). The pattern of broad H3K4me3 domains in early embryos is conserved between human and mouse. Previous results suggest that broad H3K4me3 domains in early embryos are inherited from the oocytes in mouse. We are curious about the inheritance of broad H3K27ac domains in human early embryos. Based on the following observations, we speculate that only a small part of broad H3K27ac domains in human early embryos before ZGA are inherited from oocyte, while the majority of broad H3K27ac domains are de novo established after fertilization. (1) The H3K27ac signal in human oocyte is much weaker than that in human zygote, 2-cell and 4-cell embryos, but stronger than that in human 8-cell embryos (Supplementary Fig. S2e). It suggests that the broad H3K27ac domains may exist in human oocyte, but are not widely distributed in oocyte. (2) Because the H3K27ac immunostaining signal difference between oocyte and zygote in human is similar to that in mouse, the reprogramming of H3K27ac signal from oocyte to zygote may be conserved between human and mouse (Supplementary Figs. S2e, S5c). (3) We analyzed the inheritance of broad H3K27ac domains observed in mouse zygote. For broad H3K27ac domains with lengths > 10 kb in mouse zygote, only 8.5% of them are inherited from oocyte (Fig. 3d; Supplementary Fig. S6b). The majority of broad H3K27ac domains in mouse zygote are de novo established through extensive spreading of H3K27ac peaks in gametes after fertilization (Fig. 3d). Due to the scarcity of human oocyte and large amount of oocytes required for H3K27ac ChIP-seq experiments, further investigation is needed to prove our hypothesis. The mechanism of establishing broad H3K27ac remains unknown. We show that most of broad H3K27ac domains overlap with broad H3K4me3 domains in early embryos. It’s possible that the patterns of broad H3K27ac and H3K4me3 domains are deposited by an epigenetic complex.

Because the H3K27ac patterns between sperm and oocyte are different in mouse, we are curious about the allelic H3K27ac modifications in mouse early embryos. We collected the embryos from C57BL/6N females mated with PWK/PhJ males to profile H3K27ac patterns. The parental alleles were distinguished according to the single nucleotide polymorphisms (SNPs) between these two mouse strains. Our data show that maternal H3K27ac signals are much stronger than paternal H3K27ac signals in the H3K27ac-enriched regions in mouse zygote (Supplementary Fig. S14a). At the 2-cell stage and onward, the H3K27ac signals between maternal and paternal alleles tend to become comparable, although the H3K27ac signals of maternal alleles are still slightly stronger than those of paternal alleles (Supplementary Fig. S14a). Next, we identified parental allele-specific H3K27ac peaks with stringent criteria (FDR < 0.001, reads fold change > 5) (Supplementary Fig. S14b). In mouse zygote, all allelic H3K27ac peaks are maternal allele-specific. Both maternal and paternal allele-specific H3K27ac peaks can be observed at later stages. Many allelic H3K27ac peaks locate in promoters (Supplementary Fig. S14c). Compared to allelic H3K27ac peaks, the non-allele-specific H3K27ac peaks are more enriched in promoters (Supplementary Fig. S14c). We investigated the parental gene expression of the genes whose promoters harbor allelic H3K27ac signal. Consistently, the genes with maternal allele-specific H3K27ac in promoters tend to exhibit maternally biased expression, the genes with paternal allele-specific H3K27ac in promoters tend to exhibit paternally biased expression (Supplementary Fig. S14d). Our data also show that the genes whose promoters harbor maternal allele-specific H3K27ac are involved in cell cycle regulation, DNA repair and RNA splicing (Supplementary Fig. S14e). In contrast, the genes whose promoters harbor paternal allele-specific H3K27ac are enriched in organ development (Supplementary Fig. S14f). For the distal regions with allelic H3K27ac, we compared the allelic H3K27ac peaks with the reported allelic ATAC peaks in mouse 4-cell embryos^42^. Among 86 maternal allele-specific ATAC peaks, 12 peaks show maternal allele-specific H3K27ac signal. Among 238 paternal allele-specific ATAC peaks, 28 peaks show paternal allele-specific H3K27ac signal. We also compared the allelic H3K27ac signal with germ-line imprinting control regions (ICR)^6^. Our data show that the ICR of a maternally expressed imprinted gene (*Meg3*) shows maternal allele-specific H3K27ac, and 5 paternally expressed imprinted genes show paternal allele-specific H3K27ac, such as *Snrpn* (Supplementary Fig. S14g).

Transposons play important roles in shaping our genome. Previous studies have shown that epigenetic information of DNA methylation, piRNAs and H3K9me3 is involved in the silence of transposons. Until now, it remains unknown whether any epigenetic modifications can serve as activator for transposon expression. Here we report that H3K27ac serves as an active indicator for transposon expression. Our study reveals that the establishment of H3K27ac signal in transposons is associated with the active transcription of transposons (Supplementary Fig. S13j). It is interesting that H3K9ac does not co-exist with H3K27ac and H3K18ac, specifically on the ERVKs. In a previous work^40^, the authors have demonstrated that the ZZ-type zinc finger (ZZ) of P300 functions as a reader of histone H3. The histone binding function of ZZ is essential in catalytic activities of P300. In particular, recognition of H3 by the ZZ domain of P300 promotes acetylation of primarily histone H3K27 and H3K18 sites, but not H3K9 and H3K4. It is possible that some factors can mediate the binding of P300 to H3 in ERVK elements by ZZ domains, which can further promote the acetylation of H3K27 and H3K18, but not H3K9 on ERVKs. It is also possible that comparing to the acetylations on H3K18 and H3K27 sites, H3K9ac is metabolized by a distinct set of enzymes, or the H3K9 sites on the ERVK elements are not accessible by the same set of enzymes.

We further compared the distributions of H3K27ac, H3K18ac and H3K9ac across the entire genome in mouse early embryos. The results show that many genomic regions show triple or double types of acetylations (Supplementary Fig. S15a). For H3K27ac-marked regions, 34.7%–52.3% of them are H3K27ac-specific, while the regions also harboring H3K18ac signal are more than those also harboring H3K9ac signal (Supplementary Fig. S15a). We next investigated the genomic distribution of regions with different types of acetylations. The regions with triple acetylations or H3K27/K9 double acetylations are more likely to be located in promoters than the regions with the other types of acetylations (Supplementary Fig. S15b). The majority of solo-H3K27ac regions are located in distal regions (Supplementary Fig. S15b). Next, we assessed the promoters with different types of acetylations. The results show that the genes whose promoters harbor H3K27/K18/K9 triple acetylations show the highest expression levels, and the genes with H3K27/K9 double acetylations in promoters show the second-highest expression levels (Supplementary Fig. S15c). The genes with triple or double acetylations in promoters tend to function in RNA processing and cell cycle regulation. In contrast, the genes with solo acetylation tend to participate in development of organ system (Supplementary Table S5). The acetylation patterns in promoters are dynamic with both gain and loss of acetylations during mouse early embryo development, suggesting that they may play important roles in stage-specific gene expression (Supplementary Fig. S15d). More than a half of promoters with H3K27/K18/K9 triple acetylations can maintain the triple acetylation state during development, which is consistent with the fundamental functions for cells of the related genes (Supplementary Fig. S15d and Table S5). We also investigated the distal regions with different types of histone acetylations. The distal regions with H3K27/K18/K9 triple acetylations or H3K27/K9 double acetylations are closer to TSS than the regions with the other types of acetylations (Supplementary Fig. S16a). For the distal regions with solo acetylation, the regions with H3K27ac are closer to TSS than those with H3K18ac or H3K9ac (Supplementary Fig. S16a). We were curious about the effect of acetylations at distal regions on gene expression. The genes whose closest distal regions harbor H3K27/K18/K9 triple acetylations or H3K27/K9 double acetylations show the highest expression levels (Supplementary Fig. S16b). Moreover, the genes whose closest distal regions harbor H3K27 solo acetylation show higher expression levels than those harbor H3K18 or H3K9 solo acetylation (Supplementary Fig. S16b). It supports that H3K27ac is an active enhancer marker. Consistently, the majority of DHSs are H3K27ac-enriched regions (Supplementary Fig. S16c). No more than 20% of DHSs harbor H3K18ac or H3K9ac but not H3K27ac (Supplementary Fig. S16c). Our data also show that the acetylations at distal regions are dynamic during mouse early embryo development (Supplementary Fig. S16d).

Taken together, we provide a valuable resource for the research of early embryogenesis in human. Our data demonstrate that broad domains and canonical peaks of histone modifications are associated with the expression of genes and transposons at different developmental stages during mammalian embryogenesis.

## Materials and Methods

### Human embryo collection

The regulatory framework about the use of human gametes and embryos for this research is based on the policies of the Human Biomedical Research Ethics Guidelines (set by National Health Commission of the People’s Republic of China on Dec. 1^st^, 2016), the 2016 Guidelines for Stem Cell Research and Clinical Translation issued by the International Society for Stem Cell Research (ISSCR) and the Human Embryonic Stem Cell Research Ethics Guidelines (set by China National Center for Biotechnology Development on Dec. 24th, 2003). These policies and guidelines permit human gametes, and/or human embryos created or genetic manipulated in vitro no more than 14 days, can be used specifically for scientific researches.

The aim and protocols of this study have been reviewed and approved by the Institutional Review Board of Beijing Institute of Genomics and China National Center for Bioinformation (#2016S005) and the Institutional Review Board of Reproductive Medicine, Shandong University (#2017LSZ38 and #2017LSZ39). Oocytes donated from patients taking in vitro fertilization treatments were fertilized using donated sperm by intracytoplasmic sperm injection (ICSI). Written informed consent was obtained from all oocyte and sperm donors, respectively. Human embryos are allowed to be manipulated by siRNA injection. All embryos are allowed to be cultured no more than 7 days and can be only used for molecular research analyses.

Immature MI oocytes were donated from patients who were taking in vitro fertilization (IVF) treatments. They were informed that the donation would not affect their IVF process. Those clinically useless MI oocytes were kept in in vitro maturation (IVM) medium at 37 °C in an atmosphere with 5% CO2 for 18–24 h. The IVM medium consists of M199 medium (Gibco, 11-150-059) with 20% Systemic Serum Substitute (Irvine Scientific, 99193) and 75 mIU/mL of recombinant follicle stimulating hormone (Merck Serono). The in vitro matured MII oocytes were fertilized using donated sperm by ICSI. Then, the fertilized zygotes were sequentially cultured in G1.5 medium (Vitrolife) in a humidified atmosphere at 37 °C with 6% CO_2_ in air. Cultured 2-cell, 4-cell, 8-cell embryos, morula and morphological AA grade blastocysts were collected and vitrified around 27 h, 48 h, 3 day, 4 day and 5 day after fertilization, respectively. Only high quality embryos were selected and collected according to the following morphological criteria^43^: 2-cell embryos with 2 symmetric blastomeres of equal size and with negligible fragmentation; 4-cell embryos with 4 blastomeres of equal size and with negligible fragmentation; 8-cell embryos with 8 blastomeres of equal size and without cytoplasmic fragments; morulae are compacted with > 16 cells; blastocysts with clearly visible blastocyst cavity, well developed ICM with many tightly packed cells, smooth trophoectoderm and thin zona. The embryo vitrification was carried out as described^44^. Briefly, the embryos were incubated in Vitrification Solution 1 consisting of 8% ethylene glycol and 8% dimethyl sulfoxide in Cryobase (10 mM HEPES-buffered media containing 20 mg/mL human serum albumin and 0.01 mg/mL gentamicin) at room temperature for 11 min. After initial shrinkage, embryos with original volume were transferred into Vitrification Solution 2 (16% ethylene glycol, 16% dimethyl sulfoxide and 0.68 M trehalose in Cryobase) for 1–1.5 min. Embryos were finally transferred onto Cryotop strip in an extremely small volume of solution (< 0.1 μL) and plunged into liquid nitrogen. After addition of the protective cover, the Cryotop was transferred into liquid nitrogen for storage.

The vitrified embryos on Cryotop strip were thawed rapidly by taking them from the liquid nitrogen after removal of the protective cover, then immersed in 2.5 mL of 37 °C Warming Solution 1 (1 M trehalose in Cryobase) for 1 min on a heated stage. Embryos were then transferred to 0.5 mL of Warming Solution 2 (0.5 M trehalose in Cryobase) for 3 min, and then placed into 0.5 mL Cryobase for 5 min followed by fresh 0.5 mL Cryobase for 1 min. Embryos were finally transferred to G1.5 or G2 medium (Vitrolife) to evaluate embryo quality.

The thawed embryos with high quality were picked randomly for experimental groups. Zona pellucida was removed by mechanical dissection with a glass needle. Embryos were washed several times by gentle pipetting with a narrow-bore glass pipette to remove the attached cumulus or polar bodies. After washing with 0.1% BSA/PBS for 3 times, embryos were used for ChIP-seq experiments. The numbers of embryos at different developmental stages used for ChIP-seq were shown in Supplementary Table S1. At least two biological replicates were done for each embryonic stage.

The patients with multifetal pregnancy after assisted reproductive technology treatment would receive reduction surgery at 6–8 weeks of gestation to decrease the abortion risk at middle or late pregnancy. The 6-week human embryos were collected after signed informed consents by patients. The methods for reduction surgery were referred from previous study^45^. The samples were washed carefully in PBS to remove blood and other contaminations completely. The embryos were cut into pieces for ChIP-seq experiments. Two 6-week embryos were used for two biological replicates.

### Mouse embryo collection

Wild-type 6–8 weeks old female C57BL/6N mice and male C57BL/6N or PWK/PhJ mice (Beijing Vital River Laboratory Animal Technology, Beijing, China) in healthy status were used in this study. They were housed under a 12 h light/dark cycle in specific pathogen-free conditions. The husbandry and experimental procedures were carried out according to the guidelines of the Institutional Animal Care and Use Committee of Institutes of Genetics and Developmental Biology, Chinese Academy of Sciences.

To induce ovulation, female mice were injected intraperitoneally with pregnant mare serum gonadotropin (PMSG, 5 IU) followed by human chorionic gonadotropin (hCG, 5 IU) injection 48 h later. Mouse PN5 zygotes, late 2-cell, 4-cell, 8-cell, morula and blastocyst stage embryos were collected at 27 h, 43 h, 54 h, 68 h, 79 h and 92 h post hCG administration, respectively. To collect MII oocytes, the female mice were killed at 20 h after hCG injection without mating. The oviducts were clipped mechanically with a razor blade to release oocytes. The oocyte-granulosa cell complexes were transferred into M2 medium (Sigma, M7167) containing 0.3 mg/mL hyaluronidase to remove the granulosa cells. The oocytes were further washed several times with M2 medium.

Zona pellucida was removed by exposure to acid Tyrode’s solution (Sigma, T1788). Polar bodies were dissociated by gentle pipetting with a narrow-bore glass pipette. After washing with 0.1% BSA/PBS, embryos were used for ChIP-seq. The number of embryos used for ChIP-seq was shown in Supplementary Tables S3 and S4. At least two biological replicates were carried out.

### Gene knockdown by siRNA injection

To investigate the function of HDACs and P300 in human early embryo development, the following siRNAs were used for gene knockdown:

HDAC1 siRNA #1 (CUAAUGAGCUUCCAUACAATT)^46^,

HDAC1 siRNA #2 (GCUUCAAUCUAACUAUCAATT)^47^,

HDAC2 siRNA #1 (UCCGUAAUGUUGCUCGAUGTT)^46^,

HDAC2 siRNA #2 (GCGGAUAGCUUGUGAUGAATT)^48^,

HDAC3 siRNA #1 (GAUGCUGAACCAUGCACCUTT)^46^,

HDAC3 siRNA #2 (CCAAGAGUCUUAAUGCCUUTT)^49^,

P300 siRNA #1 (CCCCUCCUCUUCAGCACCATT)^50^,

P300 siRNA #2 (CAGAGCAGUCCUGGAUUAGTT)^51^, and negative control siRNA (UUCUCCGAACGUGUCACGUTT). HDAC1 siRNA #1, HDAC2 siRNA #1, and HDAC3 siRNA #1 were pooled as HDACs siRNA #1. HDAC1 siRNA #2, HDAC2 siRNA #2, and HDAC3 siRNA #2 were pooled as HDACs siRNA #2. 6.6 μM HDACs siRNA #1 or #2 (the concentration of each HDAC component was 6.6 μM) and 10 μM P300 siRNA or negative control siRNA were used for microinjection. The donated IVM oocytes were in vitro fertilized and cultured in G1.5 medium as described above. The siRNA solution was loaded into injection pipette and injected into zygotes before pronuclear fading using Eppendorf PiezoXpert and Eppendorf CellTram vario microinjector. The injected embryos were cultured in G1.5 medium (Vitrolife) in a humidified atmosphere at 37 °C with 6% CO2 in air. The embryos with normal morphology were harvested at appropriate time. The embryos injected with HDACs siRNA were harvested at 8-cell stage, while the embryos injected with P300 siRNA were collected at morula stage. One HDACs siRNA #1-injected 8-cell embryo, one HDACs siRNA #2-injected 8-cell embryo, two negative control siRNA-injected 8-cell embryos, one P300 siRNA #1-injected morula, two P300 siRNA #2-injected morulae and three negative control siRNA-injected morulae were collected for RNA-seq. Four P300 siRNA #1-injected morulae and four P300 siRNA #2-injected morulae were collected for H3K27ac ChIP-seq (Supplementary Table S2).

To examine the role of P300 in mouse early embryo development, the in vitro fertilized zygotes from C57BL/6 mouse were injected with 5 μM negative control or P300 siRNA at 7 h post insemination. The sequences of P300 siRNAs used for mouse assays were listed as following: mouse P300 siRNA #1: GGUAUGAUGAACAGUCCAGTT^52^; mouse P300 siRNA #2: GGACUACCCUAUCAAGUAATT^53^. The P300 KD zygotes were collected at 8 h post injection. Four control siRNA-injected mouse 8-cell embryos, two P300 siRNA #1-injected 8-cell embryos, two P300 siRNA #2-injected 8-cell embryos, two P300 siRNA #1-injected 4-cell embryos and two P300 siRNA #2-injected 4-cell embryos were collected for RNA-seq (Supplementary Table S2). The P300 KD zygotes and 4-cell embryos were also collected for H3K27ac ChIP-seq (Supplementary Table S3). All the siRNAs used in this study were synthesized by GenePharma company.

### SAHA treatment for human embryos

For SAHA treatment, the donated IVM human oocytes were in vitro fertilized and cultured in G1.5 medium. The zygotes were then cultured in G1.5 medium containing 10 uM SAHA (Selleck, S1047) in a humidified atmosphere at 37 °C with 6% CO2 in air. The 8-cell embryos with normal morphology were harvested at 3 days post fertilization. Two SAHA-treated 8-cell embryos and two control 8-cell embryos were collected for RNA-seq.

### α-amanitin and SAHA treatment for mouse embryos

For α-amanitin treatment, mouse zygotes (26 h post hCG administration) were cultured in EmbryoMax KSOM medium (Millipore, MR-106-D) containing 25 ng/μL α-amanitin (Cayman, #17898) or 10 μM SAHA (Selleck, S1047). The treated embryos were collected at the late 2-cell stage (48 h post hCG administration). The α-amanitin and SAHA-treated late 2-cell embryos were used for H3K27ac ChIP-seq assays (Supplementary Table S3).

### Low-input ChIP-seq

The collected human and mouse embryos were cross-linked in 10 μL 1% formaldehyde in PBS at room temperature for 10 min. 2.28 μL 1.25 M glycine solution was added, mixed by gentle tapping and incubated at room temperature for 5 min. The cross-linked samples were then stored at –80 °C.

5 μL Dynabeads Protein A beads (Life Technologies, 10001D) were washed twice with 150 μL ice-cold RIPA buffer (10 mM Tris-HCl, pH 7.5, 140 mM NaCl, 1 mM EDTA, 0.5 mM EGTA, 1% Triton X-100, 0.1% SDS, 0.1% Na-deoxycholate, 1 mM PMSF, 1× Cocktail proteinase inhibitor, 20 mM Na-butyrate) and resuspended in RIPA buffer to a final volume of 150 μL in a 200 μL tube. 1.5 μL antibody was added into the bead suspension, then incubated on a tube rotator for at least 3 h at 4 °C. The antibodies used in this study include anti-H3K27ac antibody (Abcam, ab4729), anti-H3K4me3 antibody (Abcam, ab8580), anti-H3K9ac antibody (Active Motif, 39917), anti-H3K18ac antibody (Abcam, ab1191) and anti-H3K9me3 antibody (Active Motif, 39162). The antibody-coated beads were washed twice in RIPA buffer, then suspended with 110 μL RIPA buffer for further use.

The cross-linked samples were incubated in 120 μL lysis buffer (50 mM Tris-HCl, pH 8.0, 10 mM EDTA, pH 8.0, 0.2% SDS, 1 mM PMSF, 1× proteinase inhibitor cocktail, 20 mM Na-butyrate) for 20 min on ice, then sonicated using Diagenode Bioruptor sonication device for 10 cycles by 30 s ON and 30 s OFF. After centrifugation at 12,000× *g* for 10 min at 4 °C, 12 μL of the supernatant was taken out for input. The left supernatant was transferred to the 200 μL tube containing 110 μL suspended antibody-coated Protein A beads, then incubated on a tube rotator overnight at 4 °C.

For human 2-cell, 4-cell embryos and mouse zygote, the incubated Protein A beads were washed by RIPA buffer with 300 mM NaCl for twice, RIPA buffer with 500 mM NaCl for twice, and TE buffer (10 mM Tris-HCl, pH 8.0, 1 mM EDTA) for one time. For embryos at other stages, the incubated Protein A beads were washed by RIPA buffer with 300 mM NaCl for four times, and TE buffer for one time. After that, the beads were transferred to a new 200 μL tube, then incubated in 100 μL ChIP elution buffer (10 mM Tris-HCl, pH 8.0, 5 mM EDTA, 300 mM NaCl, 0.5% SDS) containing 2.5 μL proteinase K (Qiagen, 20 mg/ml stock) at 55 °C for 1 h, 65 °C for 6 h.

Eluate was transferred to a 0.6 mL tube. A second elution with 20 μL ChIP elution buffer was performed for 5 min and pooled with the first eluate. ChIP DNA was purified by 1.8 volume SPRIselect beads (Beckman Coulter, B23318), then dissolved in 50 μL TE buffer.

NEBNext Ultra II DNA Library Prep Kit for Illumina (NEB, E7645S) was used for library construction according to manufactory’s instruction. DNA was end repaired and A-tailed by adding 7 μL NEBNext Ultra II End Prep Reaction Buffer and 3 μL NEBNext Ultra II End Prep Enzyme Mix. Samples were incubated in a thermal cycler at 20 °C for 30 min, 65 °C for 30 min, and finally cooled to 4 °C. Adaptor ligation was performed by adding 30 μL NEBNext Ultra II Ligation Master Mix, 1 μL NEBNext Ligation Enhancer, 0.5 μL 200 mM ATP and 2.5 μL Y-shapped Illumina Multiplexing Adaptors (1.5 μM). Samples were thoroughly mixed and incubated at 20 °C for 25 min. After adaptor ligation, 5 ng circular carrier DNA (SP64 plasmid) was added, then 1.2 volume SPRIselect beads were used to purify DNA. PCR amplification was performed with NEBNext Ultra II Q5 Master Mix. In order to obtain adequate amount of DNA for sequencing, the cycle of PCR amplification was determined according to the amount of 1 μL amplified DNA, which was evaluated using FlashGelTM System (Lonza, 57063). The volume of PCR product was adjusted to 150 μL by adding TE buffer. The 350–600 bp DNA fragments were selected with 0.5 volume plus 0.3 volume SPRIselect beads, then eluted in 15 μL water. The libraries were sequenced on Hiseq ×10 or NovaSeq with paired-end 150 bp (Illumina).

### RNA-seq library preparation

For each RNA-seq library, only one 4-cell embryo, 8-cell embryo or morula was used. SMART-Seq v4 Ultra Low Input RNA Kit for Sequencing (Takara, 634888) was used for RNA-seq library preparation according to manufacturer’s instructions. Briefly, the sample volume of siRNA-injected human embryo or SAHA-treated embryo was adjusted to 9.5 μL with nuclease-free water. After adding 1 μL 10× Reaction Buffer (0.95 μL 10× Lysis Buffer, 0.05 μL RNase Inhibitor), samples were incubated at room temperature for 5 min, then placed on ice. 2 μL 3' SMART-Seq CDS Primer II A (12 μM) was added, followed by incubation at 72 °C for 3 min. Samples were placed immediately on ice for 2 min. cDNA synthesis reaction was prepared by adding 4 μL 5× Ultra Low First-Strand Buffer, 1 μL SMART-Seq v4 Oligonucleotide (48 μM), 0.5 μL RNase Inhibitor (40 U/μL) and 2 μL SMARTScribed Reverse Transcriptase. The reaction was performed in a thermal cycler with following program: 42 °C for 90 min, 70 °C for 10 min, 4 °C forever. After adding 25 μL 2× SeqAmp PCR Buffer, 1 μL PCR Primer II A (12 μM), 1 μL SeqAmp DNA Polymerase and 3 μL nuclease-free water, 16 rounds PCR amplification was performed with following program: 95 °C for 1 min; 98 °C for 10 s, 65 °C for 30 s and 68 °C for 3 min, repeat these 3 steps for 15 times; 72 °C for 10 min; 4 °C forever. The amplified cDNA was purified by SPRIselect beads, then fragmented to 200–400 bp by Covaris sonicator (Covaris). Sequencing libraries were prepared with NEBNext Ultra II DNA Library Prep Kit for Illumina (NEB, E7645S) as described above. The libraries were sequenced on Hiseq ×10 or NovaSeq with paired-end 150 bp.

### Immunostaining

After removal of zona pellucida, human and mouse oocytes and embryos were washed three times in PBS, and fixed in 4% paraformaldehyde, 0.04% Triton, 0.3% Tween and 0.2% sucrose in PBS for 20 min at room temperature. After washing three times in PBS for 5 min, the embryos were permeabilized in PBS containing 0.5% Triton for 20 min. Embryos were blocked in 3% BSA in PBST for 2 h at room temperature, then incubated with anti-H3K27ac or anti-H3K4me3 antibodies (1:200 dilution) for about 12 h at 4 °C. After washing 6 times in PBST, embryos were blocked in 3% BSA in PBST for 30 min and then incubated for 1 h at room temperature with Alexa Fluor 488-labeled goat anti-rabbit IgG (Beyotime, P0176). Embryos were washed in PBST for 3 times, then stained with DAPI (Beyotime, C1002) for 5 min. After washing in PBST for 3 times, embryos were mounted in Prolong gold antifade reagent (Thermo Fisher, P10144). Confocal images were obtained by Zeiss LSM 710 confocal microscope using a 63× oil objective. All the staining assays were repeated independently at least twice. Images were processed and quantified by ImageJ software (version 1.53a). H3K27ac fluorescence intensity was measured by ImageJ.

### ChIP-seq data analysis

The ChIP-seq raw reads were cropped to 100 bp, and the reads with low qualities were removed by Trimmomatic v0.33^54^. Paired reads were mapped to human genome hg19 or mouse genome mm10 by Bowtie2 v2.3.1 with parameters “-X 800 --no-mixed --no-discordant”^55^. Reads with low mapping quality (MAPQ < 10) and PCR duplicated reads were removed by Samtools v1.9 and Picard v2.18.25, respectively^56,57^. The fragment per kilobase per million mapped reads (FPKM) value for non-overlapping 5 kb window on entire genome was calculated as tag density in ChIP-seq data. The Pearson’s correlation coefficient (*r*) of tag densities between two replicates were used to evaluate the reproducibility. The alignment data of two or more replicates at each stage were merged for downstream analysis.

To exclude the influences of sequencing depth on the numbers of H3K27ac peaks or domains in human early embryos, we randomly selected 13.5 millions of paired-end (PE) reads to call typical H3K27ac peaks for human embryos at each developmental stage (such as 8-cell, morula, blastocyst and 6-week stages) after ZGA, and 70 millions of paired-end reads to call broad H3K27ac domains for the 2-cell and 4-cell human embryos. Because the genomic regions covered by broad H3K27ac domains before ZGA were much larger than those covered by typical H3K27ac peaks after ZGA, we could not obtain good results by using as few as 13.5 millions of PE reads to do peak calling for embryos at the 2-cell and 4-cell stages. As a result, more paired-end reads were used to call H3K27ac broad domains at the 2-cell and 4-cell stages. Next, the peaks were called by MACS2 v2.1.0^58^. We used parameters “-p 1e-3 --broad --broad-cutoff 0.01 --max-gap 500” to call H3K27ac typical peaks or broad domains in human embryos. The H3K27ac peaks or domains with low H3K27ac signal were further filtered. The sum of FPKM values of consecutive 100 bp bins located in an H3K27ac peak or domain was referred as the signal of such peak or domain. The peaks or domains with top 75% of H3K27ac signal for human embryo at each stage were kept for downstream analysis. The remained peaks or domains within 5 kb were further merged as described^7^. For mouse ChIP-seq data analysis, 15 millions of PE reads were randomly selected to do peak calling for each sample collected after ZGA stage. The H3K27ac, H3K18ac and H3K9ac peaks were called by MACS2 with parameters “-p 1e-3 --broad --broad-cutoff 0.01 --max-gap 500”.

Because broadly distributed H3K27ac signal could result in lower FPKM values in human 2-cell and 4-cell embryos than the embryos at later stages, we further normalized the H3K27ac signal between typical peaks and broad domains by using similar strategy as described^7^. With the assumption that the H3K27ac-marked regions with the highest signal represented fully H3K27ac modified regions and had similar H3K27ac signal levels among samples, we firstly divided genome into non-overlapping 100 bp bins, then calculated FPKM values for H3K27ac in each bin for human samples. The mean value of all the 100 bp bins located in a H3K27ac peak or broad domain was referred as the H3K27ac signal for such peak or domain. We supposed that the medium H3K27ac signal value of H3K27ac peaks or domains with top 25% signal should be equal between 8-cell embryo and 2-cell or 4-cell embryos. As a result, the scale factors for H3K27ac signal at the 2-cell and 4-cell stages were calculated.

ChIP-seq signal tracks visualized in Integrative Genomics Viewer (IGV) v2.8.6^59^ or UCSC Genome browser were firstly generated by callpeak command in MACS2 with parameters “-B --SPMR”, then the input signal was subtracted by bdgcmp command in MACS2 with parameter “-m subtract”.

To calculate H3K4me3 or H3K27ac signal at promoters, the ChIP-seq FPKM value of each promoter for sample was subtracted with the FPKM value of that promoter for input. If the subtracted FPKM value is < 0, the FPKM of the promoter for that sample will be assigned as 0. To plot H3K27ac ChIP-seq signal profile around H3K27ac peaks, the region from 2 kb upstream to 2 kb downstream of each H3K27ac peak center was segmented into non-overlapping 20-bp bins, which were labeled from bin 1 to bin 200. The ChIP-seq subtracted FPKM value for each bin was calculated as described above. The average FPKM values for bins with the same index of all the peaks were used to plot ChIP-seq profile. H3K4me3 or H3K27ac ChIP-seq signal profiles around TSS were plotted with similar method. The region of TSS ± 2.5 kb was divided into 100 non-overlapping 50-bp bins. To plot heat maps of H3K4me3 or H3K27ac ChIP-seq signal at promoter region (TSS ± 2.5 kb), the subtracted FPKM value for each non-overlapping 10-bp bin on entire genome was calculated. Each promoter region was divided into non-overlapping 50-bp bins. For each 50-bp bin, the average subtracted FPKM value for the genome-wide 10-bp bins within this region was assigned to it. Those FPKM values for all the 50-bp bins were used to draw heat maps by ggplots2 package.

DiffBind v3.8.1 was used to identify downregulated or upregulated H3K27ac peaks in human morulae upon P300 KD. Only peaks with fold change > 2 and FDR < 0.05 were referred as differential peaks upon P300 KD. For P300 KD mouse embryos, only the H3K27ac peaks with fold change > 3 were identified as differential peaks.

The published H3K4me3 ChIP-seq data for human early embryos were downloaded from GSE124718^11^. The H3K27ac ChIP-seq data of human sperm were from GSE57095^60^. The H3K27ac ChIP-seq data for mouse 8-cell embryos, the H3K4me3 ChIP-seq data for mouse oocytes and 2-cell embryos were downloaded from GSE72784^7^. The H3K4me3 ChIP-seq data for mouse zygotes were downloaded from GSE71434^9^. The H3K27ac ChIP-seq data for mouse sperm and oocyte were from GSE79230^61^ and GSE112834^62^, respectively. The H3K27ac ChIP-seq data for human heart and stomach were from GSE101345 and GSE101034^63^, respectively. The H3K9me3 ChIP-seq data for mouse zygotes were from GSE97778^10^. P300 ChIP-seq data for human embryonic stem cell were downloaded from GSE29611^64^. These ChIP-seq data were analyzed as described above.

### Genomic annotation

The hg19 refGene files from UCSC Table Browser were used for genome annotations. Only protein-coding genes were selected for analysis. TSS ± 2.5 kb was defined as promoter region. Transcription terminate site (TTS) ± 2.5 kb was defined as transcription end site (TES). The genomic regions were segmented into five groups of genomic elements including promoter, TES, exon, intron and intergenic region. The ChIP-seq peak/domain was assigned to a genomic group if there was at least 150 bp overlapping between the peak/domain and any genomic element in the group. The priority ranking of genomic assignment was promoter, TES, exon, intron and intergenic region. The Bedtools v2.26.0 was used for the analysis^65^. The H3K27ac peaks located outside promoter regions were defined as distal H3K27ac peaks. If distal H3K27ac peaks were located within TSS ± 300 kb of some genes, those genes are defined as genes with distal H3K27ac peaks. To check whether the H3K27ac peaks in human embryos were experimentally validated enhancers, the experimentally validated human enhancer data (1792 enhancers) were downloaded from VISTA database^32^.

The enrichment of histone modification peaks in retrotransposons was calculated using observed versus expected probability. The observed probability was calculated using the length of the histone modification peaks that cover the related transposon regions versus the length of the total histone modification peaks, and the expected probability was calculated using the length of the total related transposon regions versus the length of the human genome. The annotation files for human and mouse transposons were downloaded from UCSC Table Browser.

### RNA-seq data analysis

For human and mouse RNA-seq data, low quality reads and adaptor sequences were removed by Trimmomatic v0.33. Then the trimmed reads were aligned to human genome hg19 or mouse genome mm10 by Hisat v2.0.4 with parameter “--dta-cufflinks”^66^. For RNA-seq data obtained by using SMART-Seq kit, only Reads 1 from paired-end sequencing were used for mapping. The FPKM value of each gene was calculated by Cufflinks v2.2.1^67^ with parameter “-u”. The reference transcript annotation of protein-coding genes was downloaded from refGene in UCSC Table Browser. For protein-coding genes with multiple transcripts, only the longest transcripts were kept. The stage-specific expressed genes were identified based on Shannon entropy score as described in the section “Identification of stage-specific putative enhancers”. The ZGA genes were defined as those significantly upregulated in human 8-cell embryo (FPKM > 1 and fold change > 3) comparing with 2-cell embryo. DESeq2 package was used for gene differential expression analysis^68^. FDR < 0.05 and fold change > 2 were used as cutoff for differentially expressed genes. RNA-seq hierarchical clustering was also carried out by DESeq2 package. RNA-seq tracks visualized in IGV were generated by bamCoverage program in Deeptools v2.5.7^69^ with parameter “--noralizeUsingRPKM”.

RNA-seq raw data for human preimplantation embryos were downloaded from GSE36552^38^. RNA-seq data for human 6-week embryos were downloaded from CRA001056 in PRJCA000248^5^. RNA-seq data for mouse early embryos were downloaded from GSE44183^70^. RNA-seq data for mouse blastocyst were downloaded from GSE66582^42^.

### Transposon expression data analysis

To calculate transposon expression levels, RNA-seq raw data for human preimplantation embryos were downloaded from GSE71318^71^; total RNA-seq data for human heart and stomach were from GSE88510^63^. The RNA-seq data for mouse early embryos from GSE44183, and the RNA-seq data for mouse blastocyst from GSE66582 were also used for transposon analysis. The RNA-seq data were analyzed as described above. To exclude the influence of the transcription of protein-coding genes on the expression analysis of transposons, the transposons located in exons were exclude for further analysis. The analyzeRepeats.pl script in HOMER software (v4.10) was used to calculate the reads counts in transposons. The reads counts were normalized by the library sizes, then further normalized by z-score. To evaluate expression level for each transposon, the FPKM value was calculated by Cufflinks. Only the expressed transposons (FPKM > 0) were used for further analysis. To find out the differentially expressed transposons in mouse P300 KD embryos, the read counts in different transposon families were analyzed by DESeq2. The transposon families with fold change > 2 and FDR value < 0.05 were referred as significantly downregulated transposon families.

### Noncoding RNA expression data analysis

To obtain lncRNA annotation, de novo transcriptome assembly was performed by Cufflinks using human early embryo RNA-seq data from GSE71318. The transcripts with single exon and those overlapping with RefSeq genes were removed. The de novo gtf file was compared to annotation file from Ensembl, 16,869 potential novel transcripts from 7813 loci were obtained. The coding potential for all new lncRNA transcripts was determined by Coding Potential Assessment Tool (CPAT). More than 99% transcripts show no protein coding potentiality. These new transcripts were then combined with lincRNA transcripts from Ensembl and Gencode V5. Finally, 30,773 lncRNA transcripts from 16,171 loci were obtained. The RNA expression levels of those lncRNA were calculated by Cufflinks.

### eRNA analysis

To identify eRNAs, the H3K27ac peaks without H3K4me3 signal in human early embryos from 8-cell stage to 6-week stage were merged. The expression levels (FPKM) of these merged H3K27ac peaks were calculated by Cufflinks as described above based on the total RNA-seq data of human early embryos from GSE71318. To exclude the influence of the transcription of protein-coding genes, the H3K27ac peaks located in the promoters and exons were excluded for further analysis. The H3K27ac peaks with RNA FPKM > 0.5 at a developmental stage were defined as eRNAs at the corresponding stage.

### DNase-seq data analysis

The processed DNase-seq data for human early embryos were obtained from CRA000297^13^, which had been deposited in the Genome Sequence Archive (GSA). To plot DNase-seq profile around blastocyst H3K27ac peaks, the region from 2 kb upstream to 2 kb downstream of each H3K27ac peak center was segmented into 200 non-overlapping 20-bp bins, which were labeled from bin 1 to bin 200. DNase-seq FPKM value for each bin was calculated. The average FPKM values for bins with the same index of all the peaks were used to plot DNase-seq profile. The promoters overlapping with DHSs were defined as promoters with DHSs. If DHSs were located within 500 bp upstream or 500 bp downstream of a H3K27ac peak, the H3K27ac peak was referred as H3K27ac peak with DHSs, or putative enhancer.

### DNA methylation data analysis

The DNA methylation data for human 8-cell, blastocyst and 6-week embryos were downloaded from CRA000114 in PRJCA000248^72^, which had been deposited in the GSA. Low quality reads were removed by Trimmomatic v0.33. The filtered reads were aligned to reference genomes by Bismark (v0.16.3) with default parameters^73^. PCR duplicates and the overlapping regions of paired-end reads were trimmed by Bismark. The methylation level of each CpG site was calculated by a custom script. The processed DNA methylation data for human 2-cell and 4-cell embryos were downloaded from GSE81233^74^. The single-cell DNA methylome data for the embryos at the same developmental stage were merged. The CpG number was counted in each assigned region by a custom script. The CpG density was defined as the average number of CpG sites per 100 bp for this region. The DNA methylation level of each promoter (TSS ± 2.5 kb) or other specified region was measured as the average DNA methylation level of all CpG sites in the region.

### Identification of PMDs

To identify PMDs in early embryos, we calculated the DNA methylation level and the number of CpGs covered for each 10-kb window on entire genome. The regions with DNA methylation levels < 0.5 and > 20 CpGs covered were selected and merged into PMDs.

### Identification of stage-specific putative enhancers

Because enhancers are the binding sites of transcription factors, most of enhancers are narrow regions in genome. To identify putative enhancers in genome, we applied stringent criteria to call narrow H3K27ac peaks with parameters “--keep-dup all -p 1e-5”. H3K27ac peaks for human 8-cell, morula, blastocyst and 6-week embryos were concatenated to get H3K27ac peak union. The distal H3K27ac peaks with nearby DHSs in the union are considered as putative enhancers. A Shannon entropy-based method was used to identify stage-specific putative enhancers as previously described^33^. For each putative enhancer, its relative H3K27ac ChIP-seq FPKM value at developmental stage i was defined as Ri = Ei / ∑ E, where Ei is the FPKM value of putative enhancers at stage i; ∑ E is the sum of FPKM values in all stages; N is the total number of developmental stages. Then the entropy score for this putative enhancer across developmental stages can be defined as H = −1* ∑ (Ri * log_2_Ri), where the value of H ranges between 0 and log_2_(N). An entropy score close to zero indicates the putative enhancer is highly stage-specific, while an entropy score close to log_2_(N) indicates that this putative enhancer is ubiquitous. The putative enhancers with *H* < 0.5 were selected as stage-specific enhancers.

The script findMotifsGenome.pl with default parameters in HOMER software (v4.10)^75^ was used to analyze transcription factors binding motifs enrichment in stage-specific putative enhancers.

### Identification of allelic H3K27ac peaks and RNA in mouse embryos

To identify allelic reads in mouse H3K27ac ChIP-seq data, the sequencing data were aligned to SNP masked mm10 genome sequences. The SNP information between C57BL/6N and PWK/PhJ strains was downloaded from Mouse Genomes Project. SNPsplit v0.3.2 was used to distinguish paternal and maternal reads in ChIP-seq data. The H3K27ac peaks with at least 10 reads with distinguishable SNPs per kb were selected to identify allelic peaks. The significance of allele bias was evaluated by Chi-square test. The total paternal and maternal reads in mouse morula and blastocyst were used as background for Chi-square test. The *P-*values were adjusted by “Benjamini-Hochberg” method. The H3K27ac peaks with adjusted *P*-values < 0.001 and the ratio of paternal reads to maternal reads or the ratio of maternal reads to paternal reads > 5 were referred as allelic H3K27ac peaks. Similarly, RNA-seq data from GSE71434 were used to analyze allelic RNA expression in mouse early embryos^9^.

### GO analysis

For distal putative active enhancers, the nearest genes within 300 kb were chosen for function enrichment analysis by GREAT program (version 3)^76^. For protein-coding genes, DAVID v6.8 was used for GO analysis^77^. For genes with allelic H3K27ac signal or different types of histone acetylations, clusterProfiler v3.10.1 was used for GO analysis^78^.

### Statistical analysis

R (version 3.6.0) was used for statistics analysis. The statistical methods used for analysis were described in the figure legends. In summary, Pearson’s correlation coefficient was used to evaluate reproducibility of replicates. Wilcoxon rank sum test was used to examine the significance for the comparisons of RNA expression, ChIP-seq signal, peak length, DNA methylation and CpG density. Wilcoxon rank sum test was carried out by the wilcox.test function in R. The unpaired two-samples *t*-test was used for RNA differential expression analysis between siRNA KD embryos and control siRNA-injected embryos. *t*-test was also used for H3K27ac fluorescence intensity comparison. No statistical methods were used to predetermine sample size.

## Supplementary information

Supplementary figures and tables

Table S5

## Data Availability

The sequencing data in this study have been deposited in the Genome Sequence Archive under the accession number HRA002355 and CRA006815 in PRJCA009410.
