# Supplementary material for: Dynamics of histone acetylation during human early embryogenesis
Source: Cell Discov. 2023 Mar 14;9:29. doi: 10.1038/s41421-022-00514-y (PMC10011383; doi:10.1038/s41421-022-00514-y)
Supplement: Supplementary file 1 — Supplementary figures and tables [file 41421_2022_514_MOESM1_ESM.pdf]

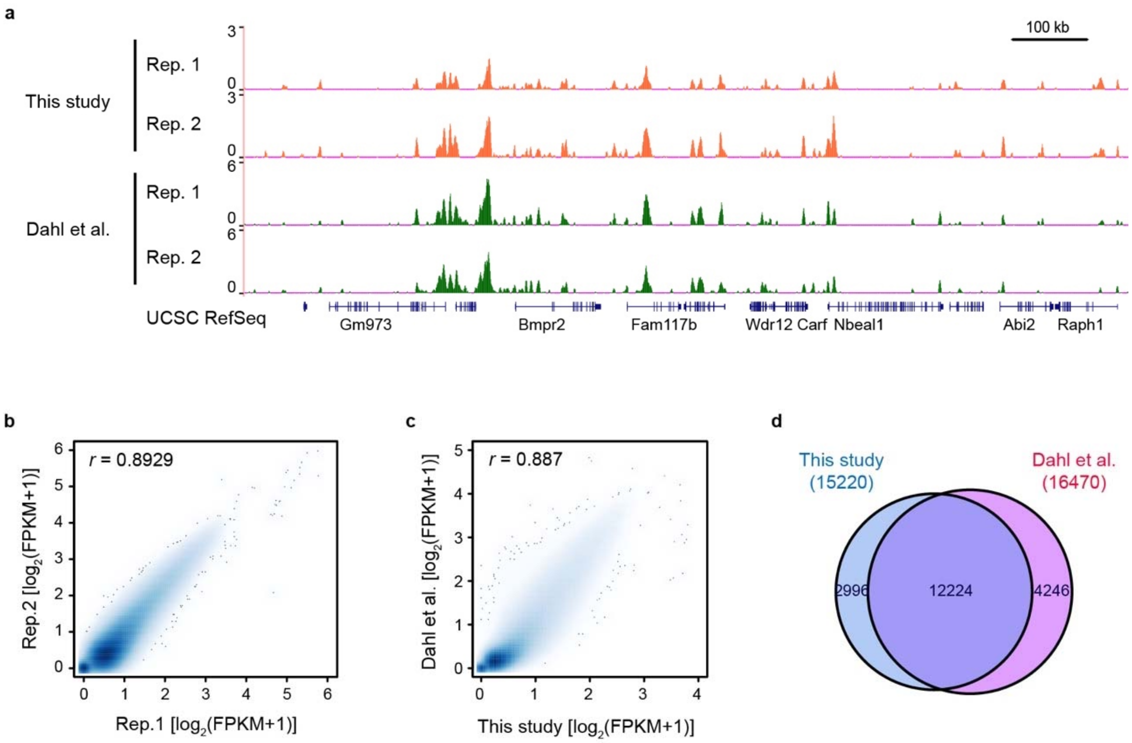

Supplementary Fig. S1 A low-input ChIP-seq method. a Genome browser snapshot of H3K27ac enrichment for mouse 8-cell embryos by a low-input ChIP-seq method using in this study. Seven mouse 8-cell embryos were used for each replicate. The published H3K27ac ChIP-seq data (Dahl et al. *Nature*. 2016) for mouse 8-cell embryos using 500 cells are used for comparison. **b** Scatter plot showing the correlation of H3K27ac signal between two replicates for mouse 8-cell embryos. The data were obtained by using the low-input ChIP-seq method. Pearson correlation coefficient (*r*) was calculated using FPKM values of each 5-kb window for the entire genome. **c** Scatter plot showing the correlation between H3K27ac signal of mouse 8-cell embryos detected by our low-input ChIP-seq method and that reported in the previous work (Dahl et al. Nature. 2016). Pearson correlation coefficient (*r*) is shown. **d** Venn diagram showing the number of overlapping H3K27ac peaks detected between our low-input ChIP-seq method and the published data for mouse 8-cell embryos.

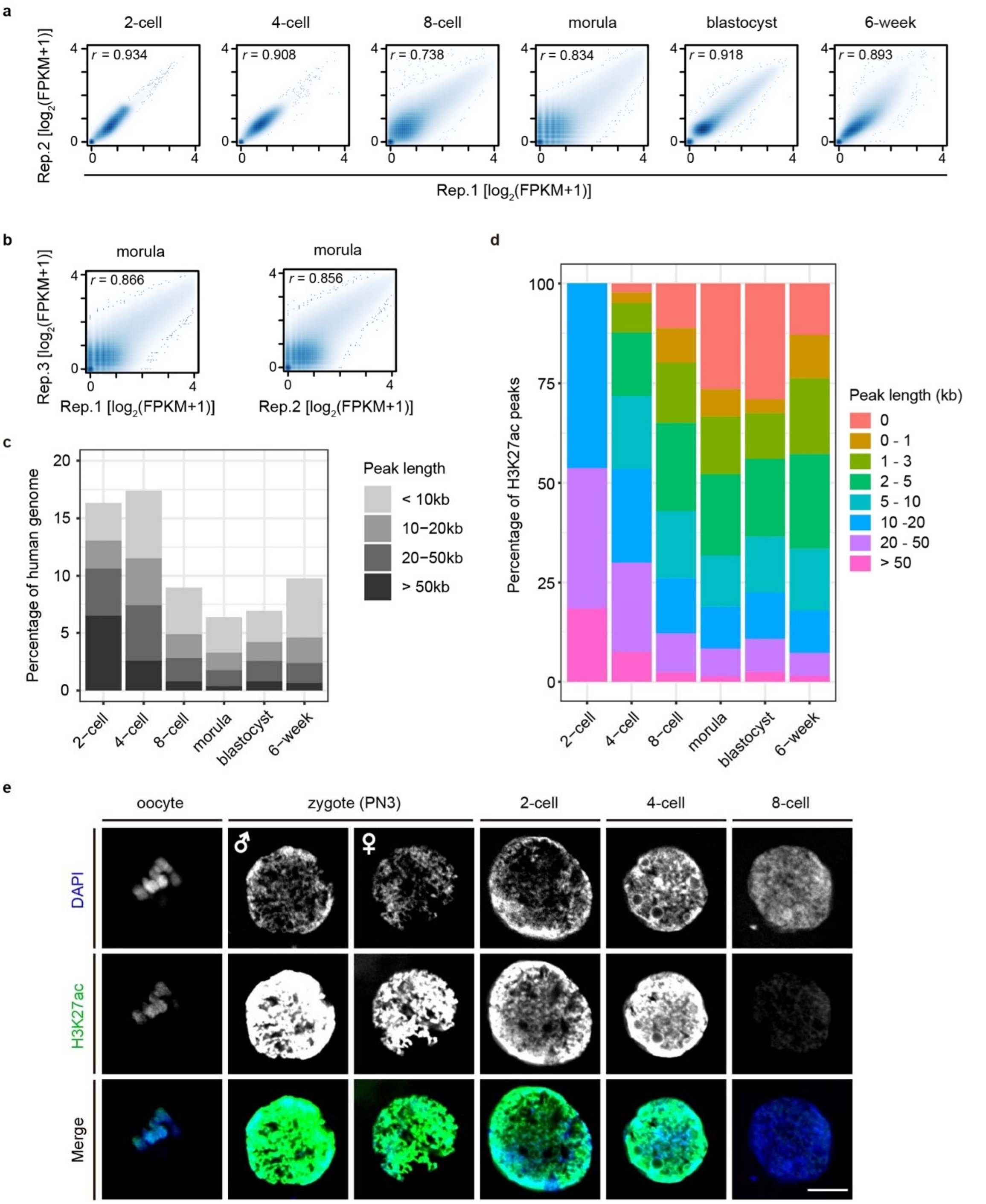

Supplementary Fig. S2 Broad H3K27ac domains in human early embryos. a Scatter plot showing the correlation of H3K27ac signal between two replicates for human early embryos. Pearson correlation coefficients (*r*) were indicated. **b** Scatter plot showing the correlation of H3K27ac signal between two replicates for human morulae. **c** Percentages of human genome covered by H3K27ac peaks with different ranges of peak lengths in human early embryos. **d** Percentages of H3K27ac peaks with different ranges of peak lengths at each stage in human early

embryos. **e** Immunostaining of H3K27ac in human oocytes and early embryos. DNA is stained by DAPI (blue). H3K27ac signal is shown in green color. The representative images of 2 independent experiments are shown. Each panel indicate the DAPI or H3K27ac signal in a nucleus of oocyte or blastomere in human. Scale bar, 10 μm.

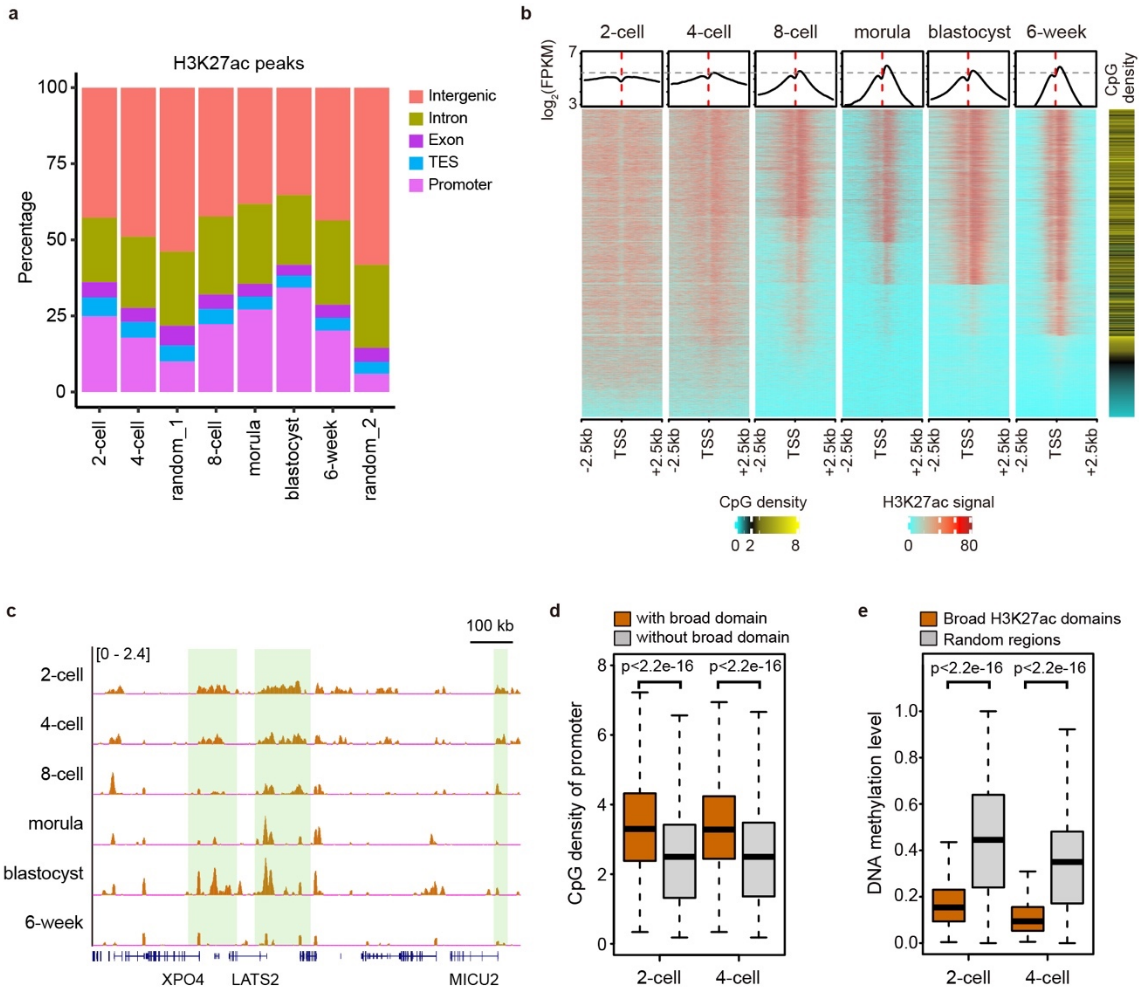

Supplementary Fig. S3 Characteristics of broad H3K27ac domains in human early embryos. **a** Percentage of H3K27ac modifications in different types of genomic elements at the indicated stages in human early embryos. Two sets of genomic regions chosen randomly which match the H3K27ac peak number and peak lengths in human 2-cell embryos (random_1) and 6-week embryos (random_2), respectively, are utilized for comparison. **b** Heat maps showing H3K27ac signal (FPKM values) at the promoters (TSS ± 2.5kb) of protein-coding genes for human embryos at the indicated stages. The genes are sorted according to the state of H3K27ac enrichment in promoters. The panels above the heat maps plot the average H3K27ac signals ($\log_2$(FPKM values + 1)) around TSS. The CpG densities of promoters are shown in the right. **c** Genome browser view of H3K27ac signal in the promoters of genes in human early embryos. Green shadows represent the locations of broad H3K27ac domains. **d** Box plots showing CpG densities of promoters with or without broad H3K27ac domains (>10kb) in human 2-cell and 4-cell embryos, respectively. Wilcoxon rank sum test is used. Boxes and whiskers represent the 25th/75th percentiles and 1.5× interquartile range, respectively. **e** Box plots comparing the DNA methylation levels between broad

H3K27ac domains and the random genomic regions in human 2-cell and 4-cell embryos, respectively. Wilcoxon rank sum test is used. Boxes and whiskers represent the 25th/75th percentiles and 1.5× interquartile range, respectively.

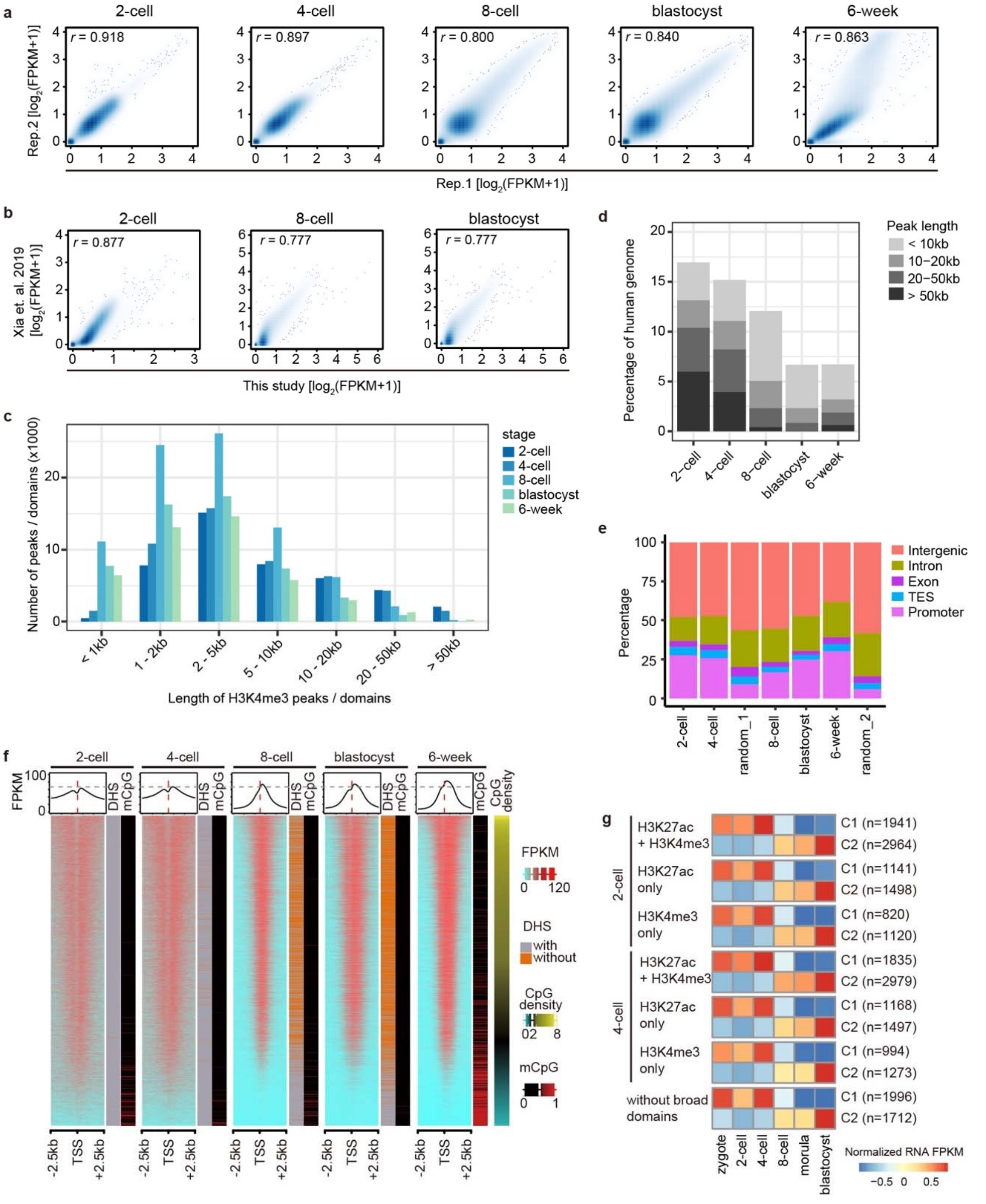

Supplementary Fig. S4 Characteristics of broad H3K4me3 domains in human embryos. a Scatter plot showing the correlation of H3K4me3 signal between two replicates for human early embryos. Pearson correlation coefficients (*r*) are indicated. **b** Scatter plot showing the correlation of H3K4me3 signal in human early embryos between this study and previously reported data (Xia et al. *Science*. 2019). Pearson correlation coefficients (*r*) are indicated. **c** Numbers of H3K4me3 peaks or domains within different ranges of lengths in human early embryos. **d**

Percentages of human genome covered by H3K4me3 peaks with different ranges of peak lengths in human early embryos. **e** Percentages of H3K4me3 peaks with different ranges of peak lengths at each stage in human early embryos. **f** Heat maps showing H3K4me3 ChIP-seq signal (FPKM values) at promoter regions (TSS ± 2.5kb) for human embryos. The chromatin accessibility states and the average DNA methylation levels of each promoter are indicated beside. Genes are sorted according to the CpG densities of promoters. The upper panels plot the average H3K4me3 ChIP-seq signals (FPKM values)) at promoter regions. **g** Heat maps showing the RNA expression dynamics of genes, whose promoters are covered by broad H3K27ac or H3K4me3 domains (>10kb) at the 2-cell and 4-cell stages, during human early embryos. At each developmental stage, the genes are classified into several groups according to broad H3K27ac and H3K4me3 signals. In each group of genes, the genes are clustered into 2 clusters (C1 and C2) by k-means method. The number of genes in each cluster is shown. The genes whose promoters are not covered by broad H3K27ac or H3K4me3 domains at both 2-cell and 4-cell stages are used for comparison.

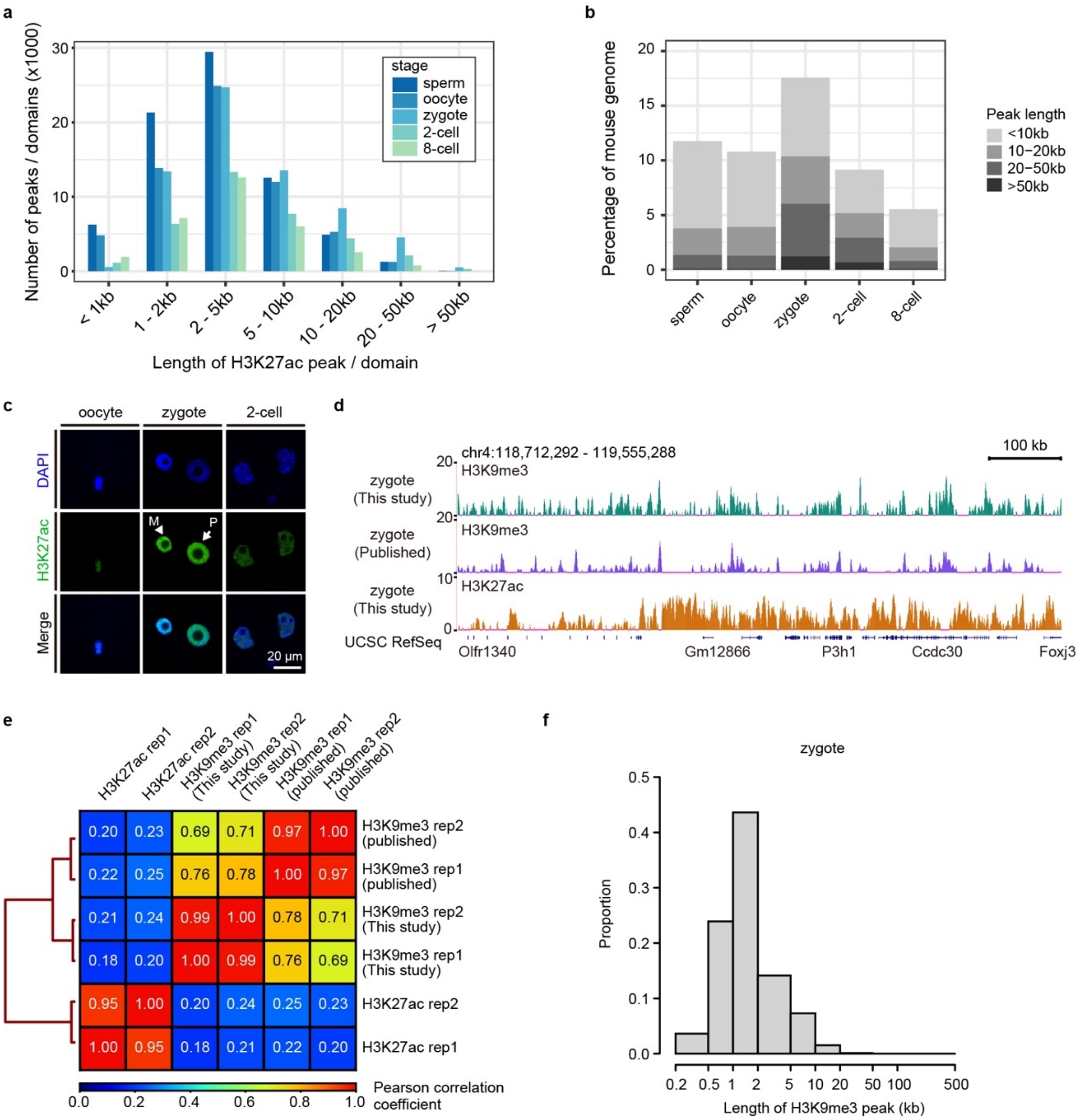

Supplementary Fig. S5 Broad H3K27ac domains are detected in mouse zygote. a Numbers of H3K27ac peaks or domains within different ranges of lengths in mouse gametes and early embryos. **b** Percentages of human genome covered by H3K27ac peaks with different ranges of peak lengths in mouse gametes and early embryos. **c** Immunostaining of H3K27ac in mouse oocytes and early embryos. DNA is stained by DAPI (blue). H3K27ac signal is shown in green color. The representative images of 3 independent experiments are shown. Scale bar, 20 μm. M, maternal pronucleus. P, paternal pronucleus. **d** Genome browser view of H3K9me3 and H3K27ac signals in mouse zygotes. The published H3K9me3 data of mouse zygotes are referred from Wang et al. *Nat Cell Biol*. 2018. **e** Hierarchical clustering of H3K9me3 and H3K27ac ChIP-seq data in mouse

zygotes according to the Pearson correlation coefficients. **f** Proportions of H3K9me3 peaks with different ranges of peak lengths in mouse zygotes.

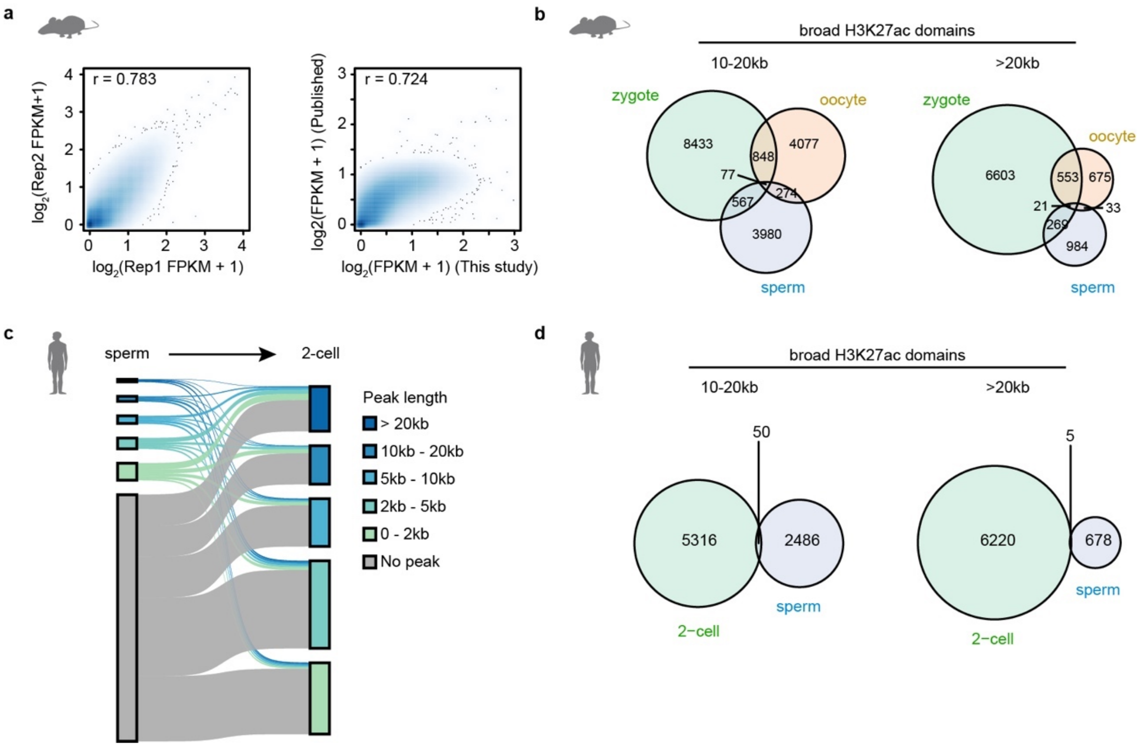

Supplementary Fig. S6 The inheritance of broad H3K27ac domains in human and mouse early embryos. a Scatter plot showing the correlation of H3K27ac signal in mouse oocytes between this study and previously reported data (Xu et al. *Nat Genet*. 2019). Pearson correlation coefficients (*r*) are indicated. **b** Venn diagrams showing the numbers of overlapping broad H3K27ac domains among zygote, oocyte and sperm in mouse. The broad H3K27ac domains are classified into two groups. The domain lengths of one group are 10-20kb, the domain lengths of the other group are > 20kb. **c** Sankey diagram showing the length dynamics of H3K27ac peaks between sperm and 2-cell embryo in human. The H3K27ac ChIP-seq data of human sperm are from Hammoud et al. *Cell Stem Cell*. 2014. **d** Venn diagrams showing the numbers of overlapping broad H3K27ac domains between 2-cell embryo and sperm in human.

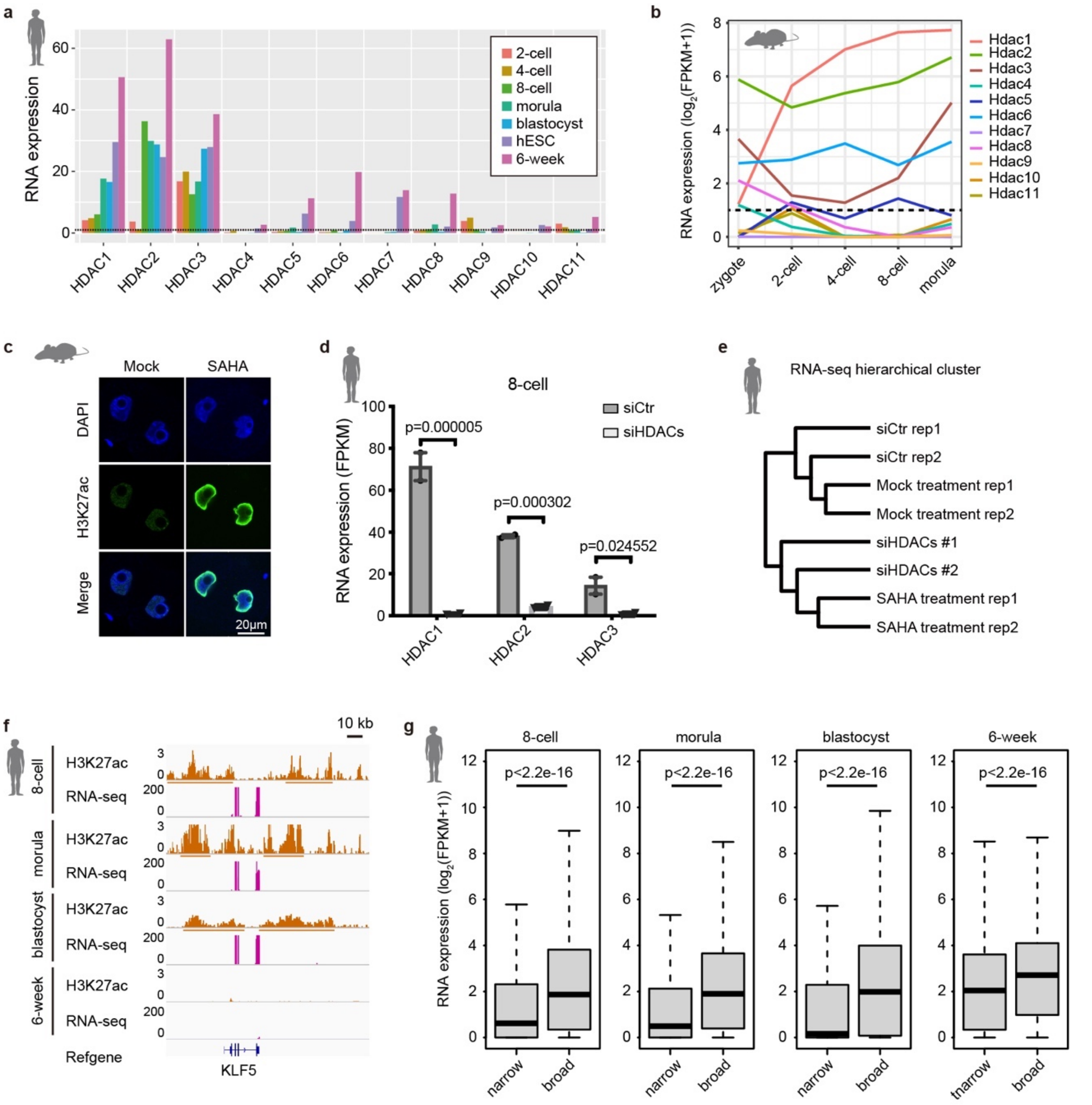

Supplementary Fig. S7 Removal of broad H3K27ac domains is associated with ZGA in human early embryos. a RNA expression levels of classical HDACs in human early embryos. The black dashed line represents the FPKM value of 1. **b** RNA expression levels of classical Hdacs in mouse early embryos. The black dashed line represents the FPKM value of 1. **c** Immunostaining images of H3K27ac signal in mouse 2-cell embryos with mock or SAHA treatment. **d** RNA expression levels of HDAC1, HDAC2 and HDAC3 for either control siRNA or HDACs siRNA injected human 8-cell embryos. siCtr represents control siRNA injected embryo (n=2). siHDACs represent HDAC1, HDAC2 and HDAC3 triple knockdown human embryos (n=2). Two different sets of triple siRNA mixtures were utilized to collect the two biological replicates of HDACs KD embryos. T-test was used. Error bars represent standard errors. **e** Hierarchical clustering results showing the relationships of RNA expression profiles among HDACs knockdown

embryos and SAHA treated human embryos based on RNA-seq results. SAHA is an inhibitor of class I HDACs, including HDAC1, HDAC2 and HDAC3. **f** Genome browser view of broad H3K27ac domains and RNA expression pattern of the associated gene in human embryo after ZGA. The locations of broad H3K27ac domains are shown below H3K27ac signal tracks. **g** Boxplots comparing RNA expression levels of narrow H3K27ac peaks (<10kb) associated genes and those of broad H3K27ac domains (>10kb) associated genes in human early embryos. Wilcoxon test is used. Boxes and whiskers represent the 25th/75th percentiles and 1.5× interquartile range, respectively.

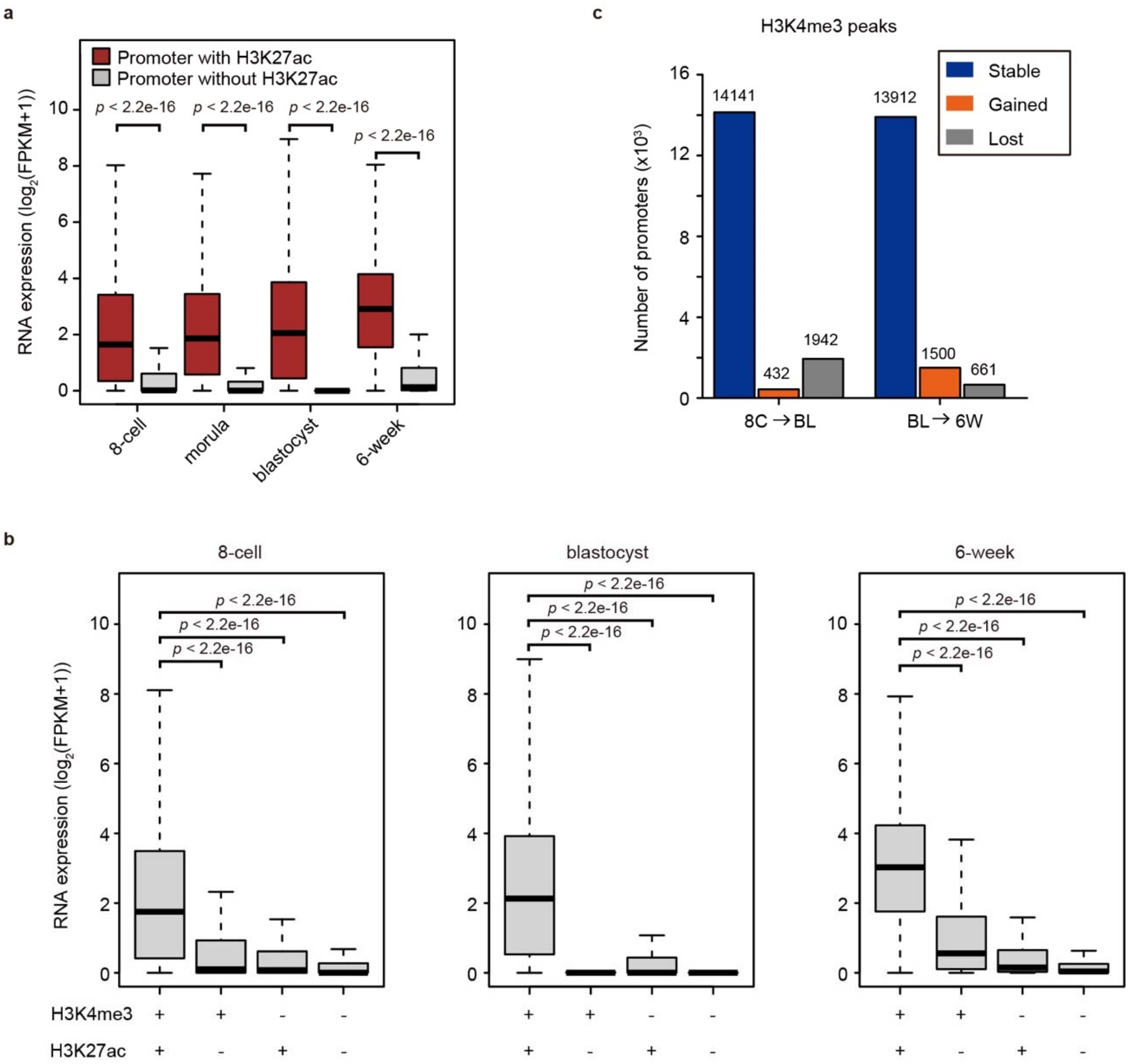

Supplementary Fig. S8 Promoter H3K27ac in human embryos. a Box plots of RNA expression levels between genes with and without H3K27ac enrichment in promoters at 8-cell, morula, blastocyst and 6-week stages. Wilcoxon test was used. Boxes and whiskers represent the 25th/75th percentiles and 1.5× interquartile range, respectively. **b** Box plots of RNA expression levels of genes with both H3K27ac and H3K4me3 enrichment at the promoters, with only H3K27ac enrichment, with only H3K4me3 enrichment, and with neither enrichment at different stages. Wilcoxon test was used. Boxes and whiskers represent the 25th/75th percentiles and 1.5× interquartile range, respectively. **c** Plot showing the dynamics of promoters with H3K4me3 enrichment between two stages. Promoter number is indicated above each bar. 8C, 8-cell; BL, blastocyst; 6W, 6-week.

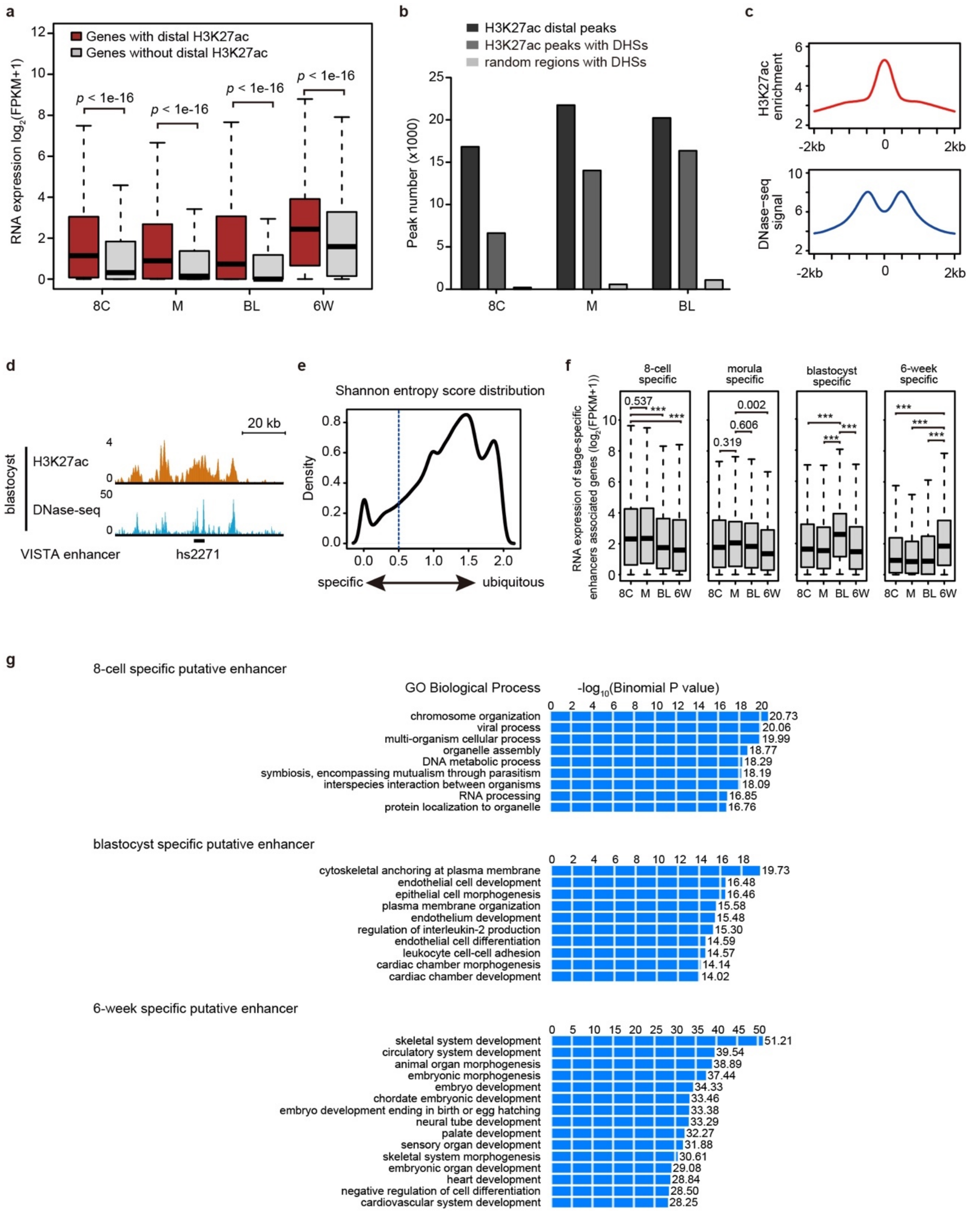

Supplementary Fig. S9 Putative enhancers in human early embryos. a Box plots of RNA expression level between genes with and without H3K27ac peaks around TSS (±300kb) at the indicated human stages. RNA expression level represents the value of $\log_2$ (FPKM +1). Wilcoxon test was used. Boxes and whiskers represent the 25th/75th percentiles and 1.5× interquartile range, respectively. **b** Bar plots indicating the number of distal H3K27ac peaks, H3K27ac peaks with DHSs, and random regions with DHSs from 8-cell stage to blastocyst

stage. If a DHS is located within a H3K27ac peak center ± 500 bp region, this H3K27ac peak is referred as a H3K27ac peak with DHSs. **c** Profiles showing the average H3K27ac enrichment and DNase-seq signal (FPKM values) around the center of H3K27ac peaks in human blastocysts. **d** Genome browser snapshot of H3K27ac modifications around VISTA enhancer hs2271 in human. The locations of enhancer element hs809 is indicated by blue box. **e** Histogram shows the distribution of the Shannon entropy score of H3K27ac enrichment levels for putative active enhancers (with both H3K27ac enrichment and DHSs) in human 8-cell, morula, blastocyst, and 6-week embryos. The H3K27ac peaks with lower Shannon entropy score are more stage-specific. The H3K27ac peaks with Shannon entropy score lower than 0.5 (dashed line) were defined as stage-specific putative enhancers. **f** Boxplots comparing RNA expression of stage-specific enhancer associated genes in morula, blastocyst and 6-week embryos. Wilcoxon test was used. *** represents $p < 0.001$. Boxes and whiskers represent the 25th/75th percentiles and 1.5× interquartile range, respectively. **g** Gene ontology (GO) analysis of genes associated with stage-specific putative enhancers.

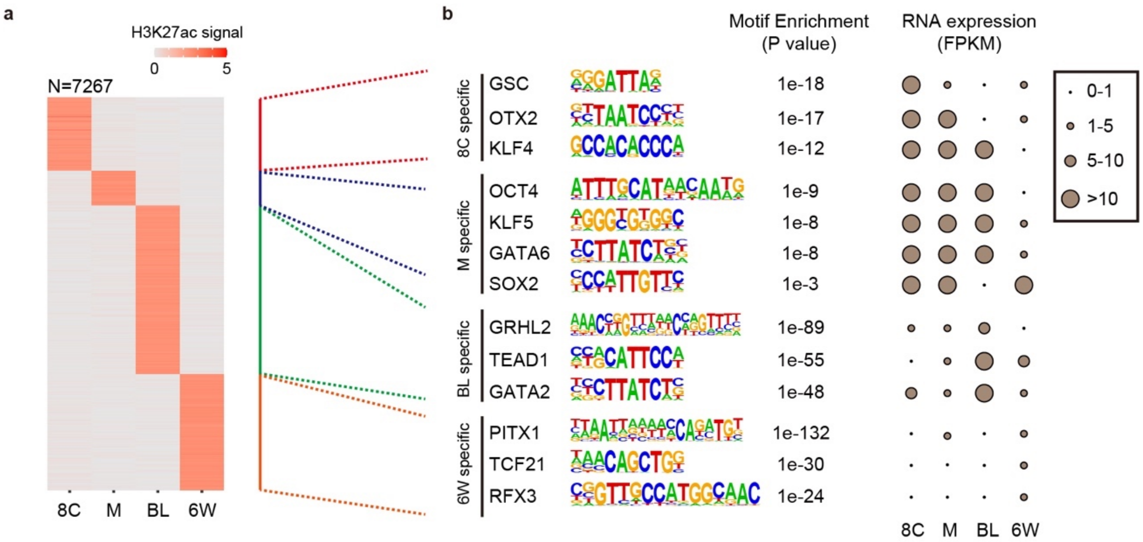

Supplementary Fig. S10 Transcription factor binding motifs enriched in stage specific putative enhancers. a Heat map showing H3K27ac ChIP-seq signal of stage-specific putative enhancers (n=7267) in human embryos. The ChIP-seq signal represents FPKM value of each enhancer. **b** Transcription factor binding motif enrichment analysis of the stage-specific putative enhancers. The binding motifs, enrichment P values, and RNA expression levels of the top enriched transcription factors at each stage are shown.

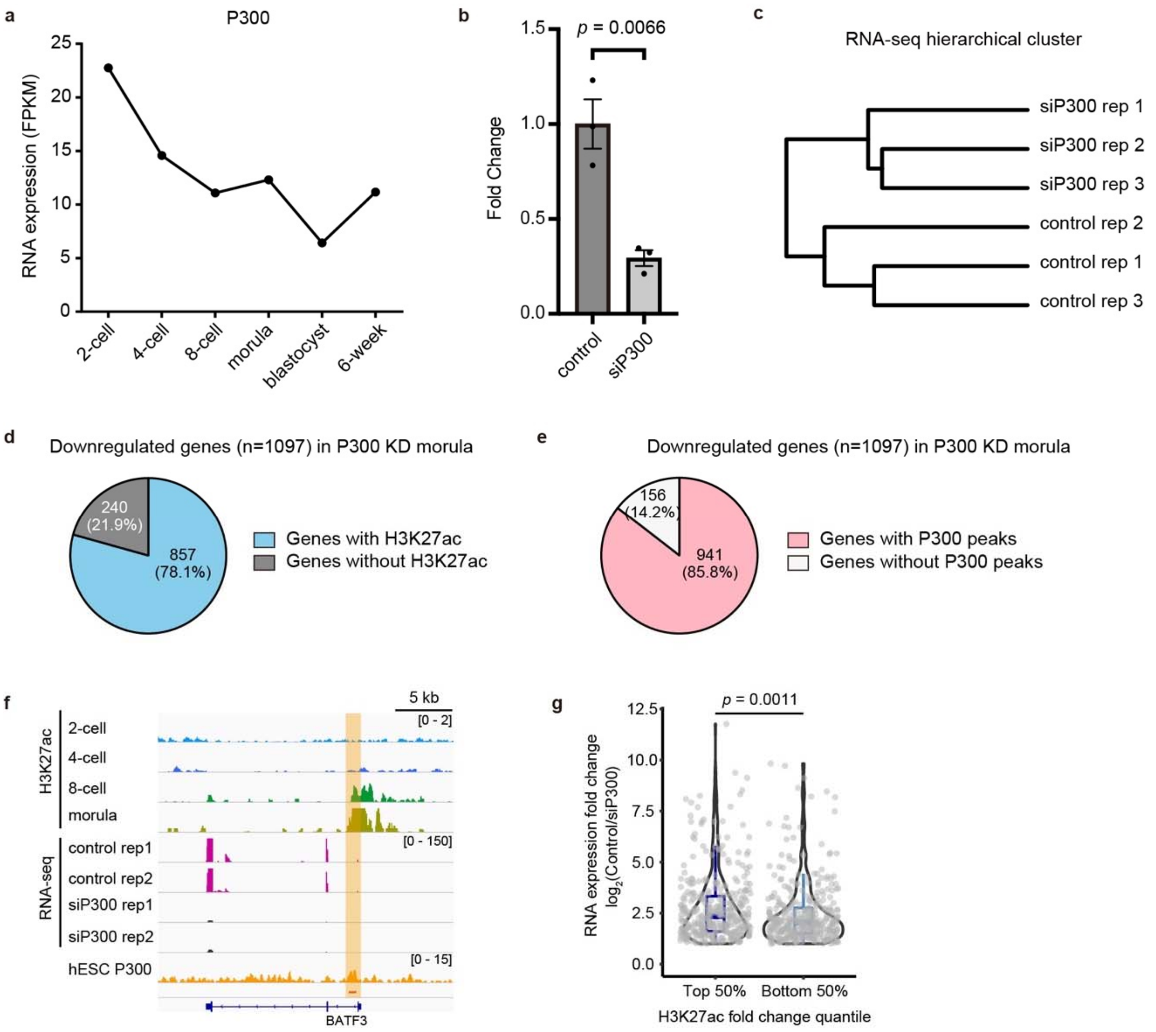

Supplementary Fig. S11 Disruption of H3K27ac in human early embryos. a RNA expression levels of P300 in human early embryos. **b** Fold change of P300 RNA expression in P300 siRNA injected human morulae comparing to control siRNA injected embryos. siCtr represents control siRNA injected embryo. Three control siRNA injected embryos, one P300 siRNA #1 injected embryo and two P300 siRNA#2 injected embryos were analyzed. T-test was used. Error bars represent standard error of mean. **c** Hierarchical clustering results showing the relationships of RNA expression profiles among control siRNA or P300 siRNA knockdown embryos and SAHA treated human embryos based on RNA-seq results. **d** Pie chart showing the numbers and percentages of genes with H3K27ac modification nearby in the downregulated genes in human P300 KD morula. **e** Pie chart indicating the numbers and percentages of genes with P300 peaks nearby in the downregulated genes in human P300 KD morula. The data of P300 peaks are from hESC. **f** Genome browser view showing H3K27ac enrichment, RNA expression pattern and P300 enrichment around gene that is downregulated in P300 KD morula. P300 peak region is shown under P300 signal track. The location

of P300 peak is labeled in shadow. **g** Violin and box plots comparing the expression fold change of H3K27ac peak associated genes (within 50kb around H3K27ac peaks) upon P300 KD between downregulated H3K27ac peaks with top 50% of fold changes upon P300 KD and the other downregulated H3K27ac peaks. Wilcoxon test was used. Boxes and whiskers represent the 25th/75th percentiles and 1.5× interquartile range, respectively.

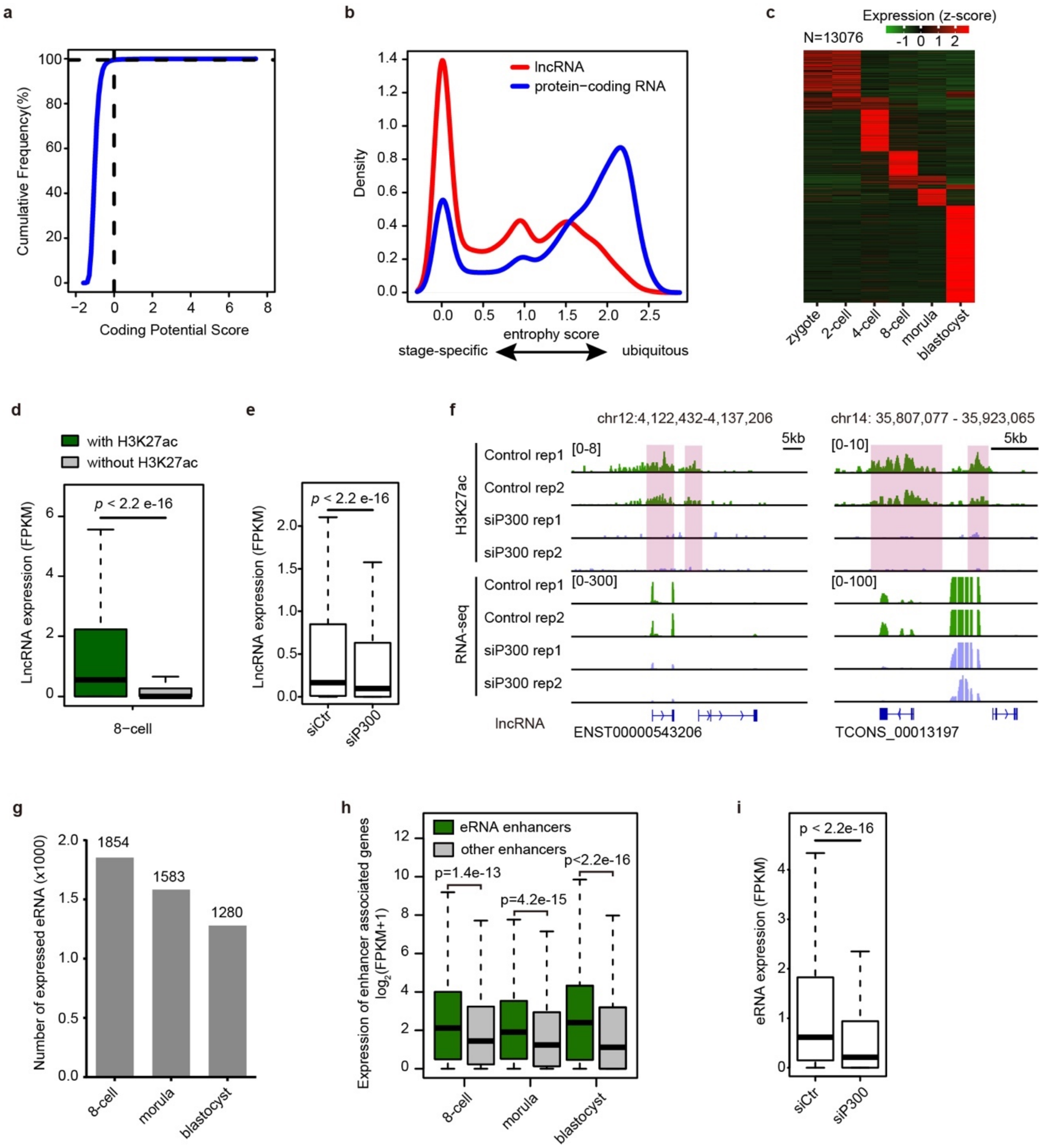

Supplementary Fig. S12 Non-coding RNAs are downregulated upon P300 KD in human morula. **a** Cumulative frequency of the coding potential score of predicted lncRNAs. lncRNAs with coding potential score < 0 means that these lncRNAs cannot encode proteins. **b** Histogram showing the distribution of the Shannon entropy scores of the expressions of protein-coding RNAs and lncRNAs in human early embryos. The RNAs with lower Shannon entropy score are more stage-specific. **c** Heat map showing the expression levels of the predicted lncRNAs in human early embryos. **d** Boxplots comparing lncRNA expression levels between those with and without H3K27ac in human 8-cell embryos. Wilcoxon test was used. Boxes and whiskers represent the 25th/75th percentiles and 1.5× interquartile range, respectively. **e** Boxplots comparing lncRNA expression levels between P300 KD morula and control morula. Wilcoxon test was used. Boxes and whiskers

represent the 25th/75th percentiles and 1.5× interquartile range, respectively. **f** Genome browser view of H3K27ac signal and RNA expression of lncRNAs in control and P300 KD morulae. The lncRNA in the right panel is a predicted lncRNA which is identified in this study. **g** Graphic indicates the numbers of expressed eRNAs in human early embryos. The expressed eRNAs are those with FPKM > 0.5. **h** Boxplots comparing the expression levels between the genes with eRNA enhancers and those with non-eRNA enhancers. Wilcoxon rank sum test is used. Boxes and whiskers represent the 25th/75th percentiles and 1.5× interquartile range, respectively. **i** Boxplots showing eRNA expression levels in P300 KD morula. Wilcoxon rank sum test is used. Boxes and whiskers represent the 25th/75th percentiles and 1.5× interquartile range, respectively.

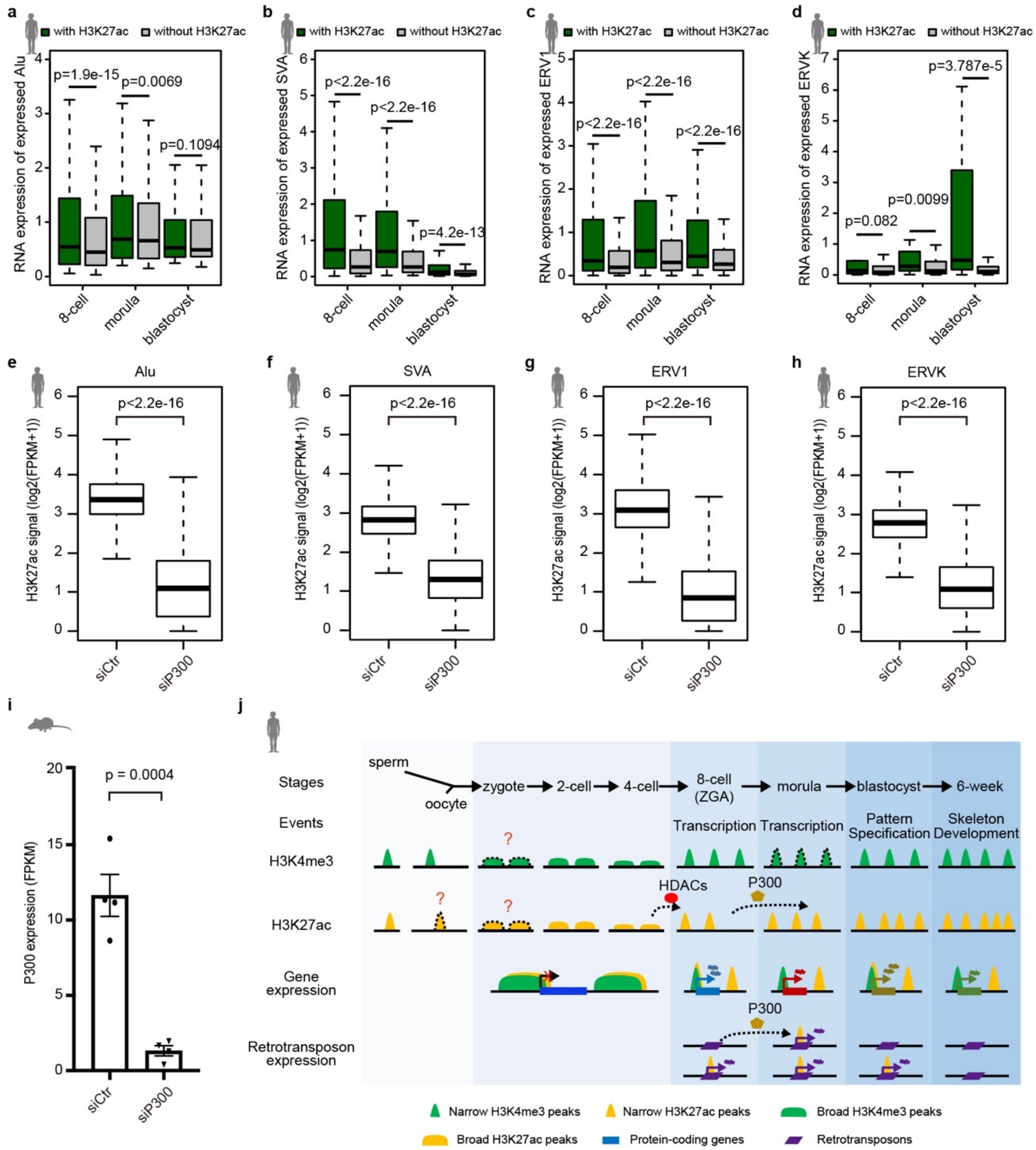

Supplementary Fig. S13 H3K27ac is essential for transposon activation in early embryos. a-d Boxplots comparing RNA expression of retrotransposons between those with H3K27ac and without H3K27ac modifications in human embryos at 8-cell, morula and blastocyst stages. The expressed retrotransposons (FPKM > 0) were analyzed for comparison. Wilcoxon rank sum test is used. Boxes and whiskers represent the 25th/75th percentiles and 1.5× interquartile range, respectively. **e-h** Boxplots showing H3K27ac signals of retrotransposons in control and P300 KD human morulae. Wilcoxon rank sum test is used. Boxes and whiskers represent the 25th/75th percentiles and 1.5× interquartile range, respectively. **i** Bar plot showing P300 expression levels in control or P300 KD mouse 8-cell embryos. Four biological replicates were performed for each group.

To collect P300 KD mouse embryos, two different siRNAs were used. Two biological replicates were collected for each P300 siRNA KD assay. T-test was used. Error bars represent standard error of mean. **j** A schematic model showing the dynamics of histone modifications during human early embryo development, and their functions in the expression regulation for gene and retrotransposon. The question marks mean that the patterns of histone modifications are not confirmed by ChIP-seq data.

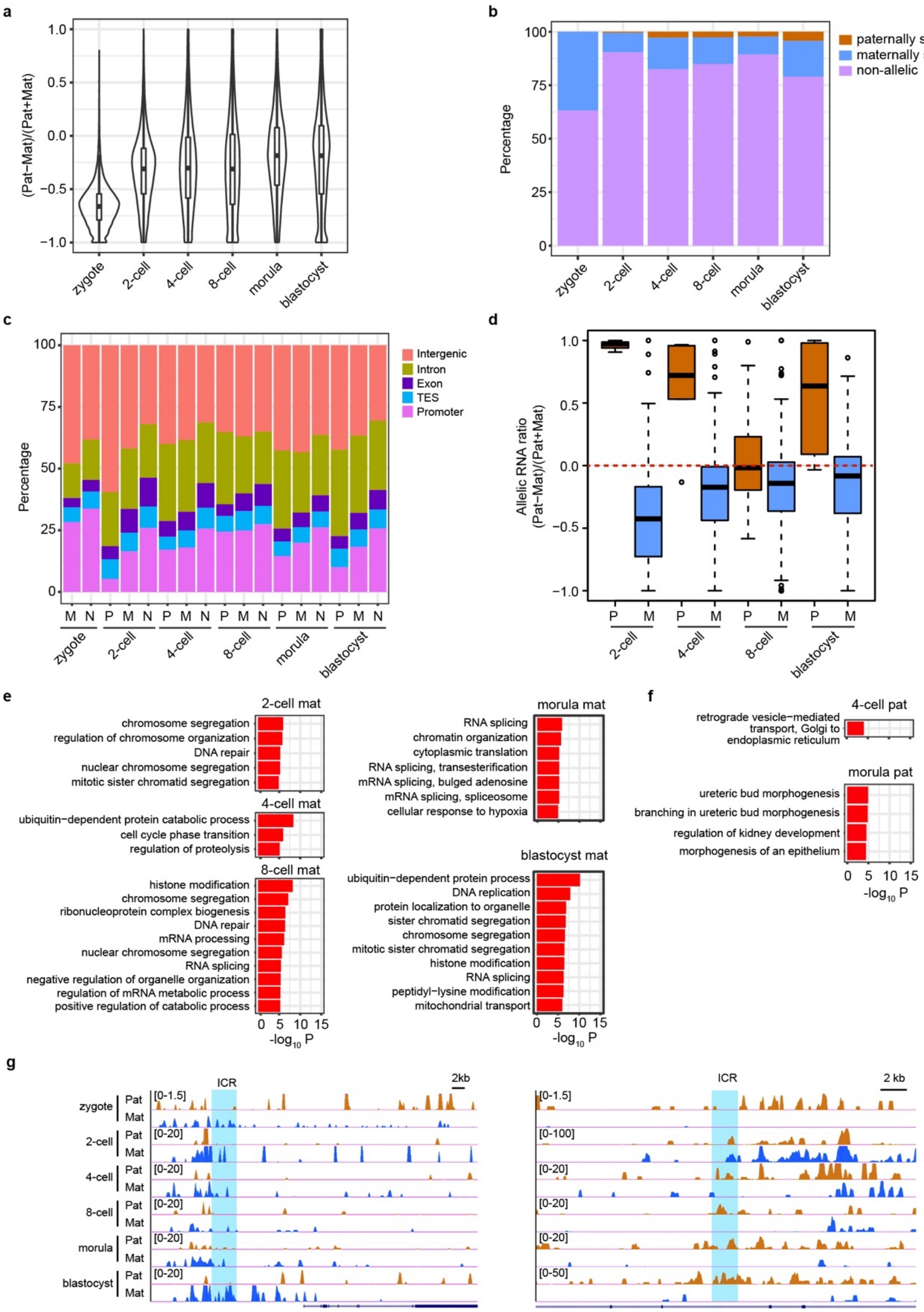

Supplementary Fig. S14 Allelic H3K27ac in mouse early embryos. a Box plot showing the allelic bias of H3K27ac peaks in mouse early embryos. The allelic bias of a H3K27ac peak is calculated by the number difference between parental reads divided by the total number of parental reads in the H3K27ac peak. **b** Percentages

of allelic H3K27ac peaks at each developmental stage in mouse early embryos. **c** Genomic distribution of allelic H3K27ac peaks in mouse early embryos. P represents paternally specific peaks, M represents maternally specific peaks, N represents non-allelic peaks. **d** Box plot showing the allelic expression bias of genes whose promoters harbor allelic H3K27ac signal in mouse early embryos. The allelic expression bias of a gene is calculated by the number difference between parental reads divided by the total number of parental reads. P represents genes with paternally specific H3K27ac signal in promoters. M represents genes with maternally specific H3K27ac signal in promoters. **e** GO enrichment of genes whose promoters harbor maternal allele-specific H3K27ac in mouse early embryos. **f** GO enrichment of genes whose promoters harbor paternal allele-specific H3K27ac in mouse early embryos. **g** Genome browser view of allelic H3K27ac signal at ICRs of imprinted genes. Meg3 is a maternally expressed imprinted gene. Snrpn is a paternally expressed imprinted gene. The ICRs are highlighted in light blue shadows.

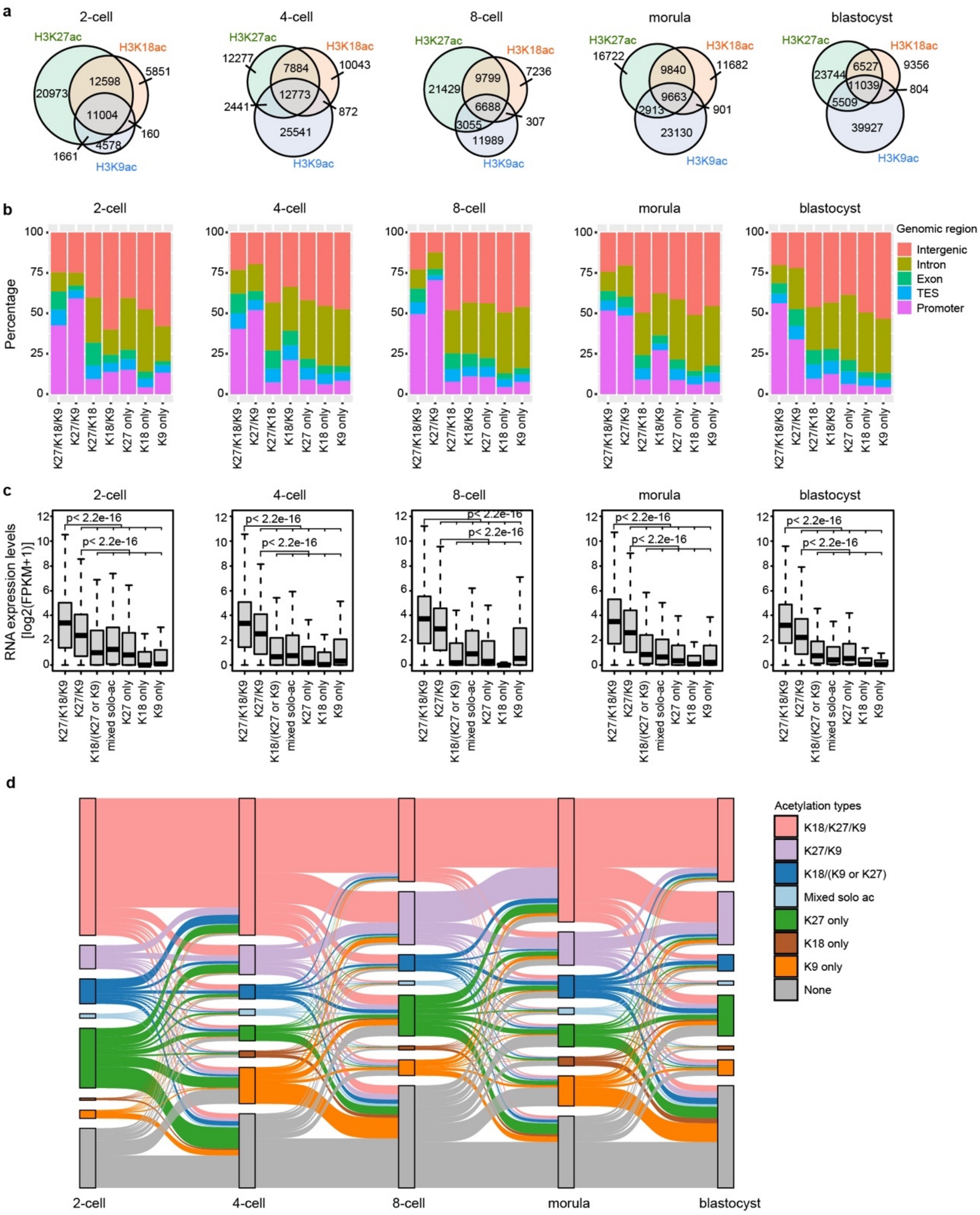

Supplementary Fig. S15 Characteristics of H3K27ac, H3K18ac and H3K9ac marked promoters in mouse early embryos. a Venn diagrams showing overlapping peaks among H3K27ac, H3K18ac and H3K9ac peaks at each developmental stage in mouse early embryos. **b** Genomic distribution of peaks with different types of histone acetylations in mouse early embryos. K27/K18/K9 represents peaks with H3K27ac, H3K18ac and H3K9ac. K27/K9 represents peaks with both H3K27ac and H3K9ac, but without H3K18ac. "K27 only" means the peaks with only H3K27ac. **c** Box plots comparing the RNA expression levels of

genes whose promoters harbor different types of histone acetylations. K18/(K27 or K9) represents the promoters with K18/K27 or K18/K9. Wilcoxon rank sum test is used. Boxes and whiskers represent the 25th/75th percentiles and 1.5× interquartile range, respectively. **d** Sankey diagrams showing the dynamics of histone acetylations in the promoters during mouse embryo development. “Mixed solo ac” represents that a promoter harbors several peaks with different types of solo acetylation.

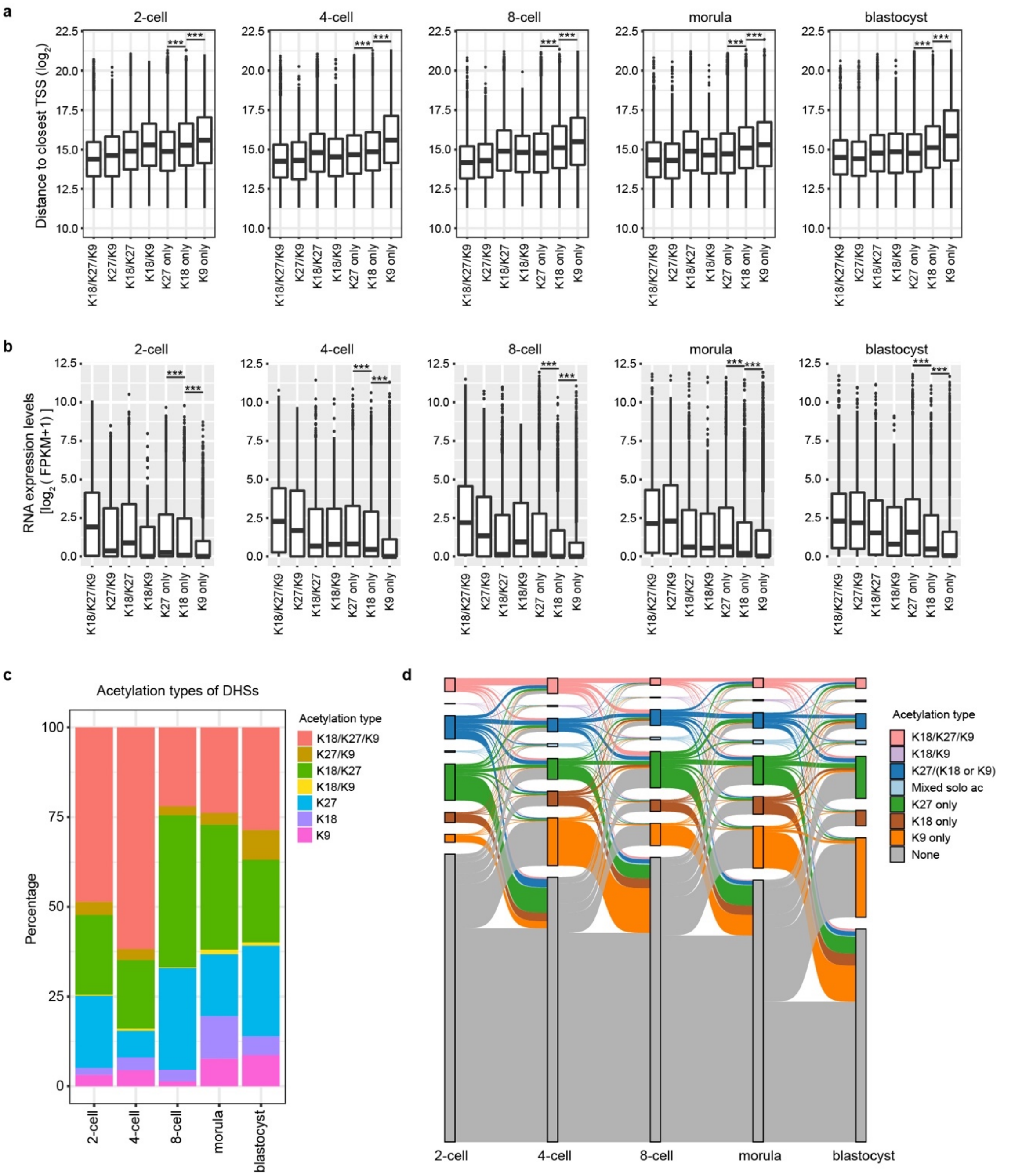

Supplementary Fig. S16 Characteristics of H3K27ac, H3K18ac and H3K9ac marked distal regions in mouse early embryos. a Box plots showing the distances of distal peaks with different types of histone acetylations to the closest TSS. Wilcoxon rank sum test is used. *** represents $p < 0.001$. Boxes and whiskers represent the 25th/75th percentiles and 1.5× interquartile range, respectively. **b** Box plots showing the RNA expression levels of genes with different types of histone acetylation at the closest distal peaks. Wilcoxon rank sum test is used. *** represents $p < 0.001$. **c** Percentages of DHSs with different types of histone acetylations in mouse early embryos. **d** Sankey diagram showing the dynamics of histone acetylations at distal peaks in mouse early embryos.

Stage	Embryo Num.	Antibody	Raw reads	Mapping rate	Unique reads
2-cell	21	H3K4me3 rep1	68,889,720	97.20%	23,101,608
	20	H3K4me3 rep2	72,214,363	97.03%	15,399,077
	22	H3K27ac rep1	64,466,642	97.68%	23,842,208
	20	H3K27ac rep2	79,697,644	97.67%	34,103,644
		Input rep1	74,812,397	96.63%	9,557,917
		Input rep2	77,947,702	96.83%	10,271,396
4-cell	13	H3K4me3 rep1	101,258,508	97.30%	14,140,608
	10	H3K4me3 rep2	88,322,922	97.24%	25,068,603
	12	H3K27ac rep1	71,051,962	97.31%	19,734,741
	10	H3K27ac rep2	76,300,861	97.47%	28,628,922
		Input rep1	77,584,929	95.63%	6,867,669
		Input rep2	90,593,288	96.31%	13,844,308
8-cell	7	H3K4me3 rep1	172,060,918	96.40%	7,494,333
	7	H3K4me3 rep2	162,169,445	96.88%	9,228,314
	7	H3K27ac rep1	81,210,168	95.66%	4,347,801
	7	H3K27ac rep2	81,501,849	96.59%	5,026,328
		Input rep1	166,012,169	97.30%	47,263,206
		Input rep2	63,877,283	96.36%	18,370,464
morula	4	H3K27ac rep1	51,217,000	89.64%	1,909,040
	4	H3K27ac rep2	64,044,795	92.05%	2,310,634
	4	H3K27ac rep3	34,923,444	94.69%	2,608,773
		Input rep1	48,839,000	96.18%	6,788,988
P300 KD morula	4	H3K27ac rep1	74,887,428	94.21%	2,263,733
	4	H3K27ac rep2	80,842,333	93.64%	3,735,113
		Input rep1	79,588,996	95.23%	7,540,944
Blastocyst	1	H3K4me3 rep1	77,929,220	96.46%	8,234,869
	1	H3K4me3 rep2	91,743,490	96.17%	8,927,474
	2	H3K27ac rep1	27,367,307	98.40%	11,261,361
	2	H3K27ac rep2	34,005,546	98.16%	17,170,732
		Input rep1	95,392,882	96.52%	17,379,662
		Input rep2	87,005,709	96.52%	10,808,524
6-week	2	H3K4me3 rep1	47,200,887	91.96%	34,257,298
		H3K4me3 rep2	42,829,428	92.48%	31,603,022
		H3K27ac rep1	46,527,636	96.71%	37,948,726
		H3K27ac rep2	44,164,322	98.23%	37,580,732
		Input rep1	34,957,041	94.79%	26,595,006
		Input rep2	50,137,777	94.64%	39,241,279

Supplementary Table S1 Sequencing and mapping information of human ChIP-seq data.

Species	Stage	Condition	Raw reads	Mapping rate	Mapping reads
Human	8-cell	Mock treatment rep1	66,205,044	96.27%	61,071,281
		Mock treatment rep2	46,753,272	94.98%	42,699,377
		SAHA treatment rep1	46,287,284	95.12%	42,729,441
		SAHA treatment rep2	52,087,886	94.61%	47,698,851
		HDACs siRNA #1	55,159,758	95.13%	51,058,654
		HDACs siRNA #2	51,408,635	95.81%	47,930,292
		Control siRNA rep1	28,449,624	94.49%	28,172,253
		Control siRNA rep2	23,676,593	95.12%	23,455,915
	morula	P300 siRNA #1	52,923,030	96.96%	50,187,681
		P300 siRNA #2 rep1	54,018,436	97.11%	51,345,032
		P300 siRNA #2 rep2	71,605,074	96.30%	71,180,393
		Control siRNA rep1	50,795,132	96.91%	48,171,637
		Control siRNA rep2	59,106,138	96.54%	55,475,220
		Control siRNA rep3	48,070,168	96.83%	47,877,686
Mouse	4-cell	Control siRNA rep1	37,468,480	90.41%	27,868,962
		Control siRNA rep2	57,451,848	90.93%	44,754,283
		Control siRNA rep3	50,703,812	83.89%	35,033,327
		Control siRNA rep4	44,923,969	85.97%	31,994,419
		P300 siRNA #1 rep1	44,382,002	87.70%	32,537,284
		P300 siRNA #1 rep2	32,376,380	91.57%	24,611,847
		P300 siRNA #2 rep1	51,164,385	91.09%	38,741,199
		P300 siRNA #2 rep2	48,656,260	83.22%	33,065,219
	8-cell	Control siRNA rep1	39,348,221	90.13%	28,717,687
		Control siRNA rep2	44,078,527	88.39%	31,433,126
		Control siRNA rep3	45,507,494	94.00%	37,318,289
		Control siRNA rep4	62,269,762	93.14%	50,286,411
		P300 siRNA #1 rep1	31,816,095	91.78%	25,316,266
		P300 siRNA #1 rep2	33,800,206	93.38%	26,933,665
		P300 siRNA #2 rep1	34,991,035	89.24%	25,295,555
		P300 siRNA #2 rep2	54,472,321	80.78%	37,272,015

Supplementary Table S2 Sequencing and mapping information of RNA-seq data with human and mouse embryos.

Stage	Strains	Emb. Num.	Antibody / treatment	Raw reads	Mapping rate	Unique read pairs
8-cell		7	H3K27ac rep1	48,677,512	97.68%	7,868,779
		7	H3K27ac rep2	43,789,438	97.63%	4,703,602
	C57xPWK*	15	H3K27ac rep3	25,437,522	95.15%	3,307,559
			Input	44,987,541	81.52%	21,851,741
oocyte		100	H3K27ac rep1	61,748,678	85.39%	2,866,210
		100	H3K27ac rep2	72,115,982	88.91%	2,386,230
			Input	61,196,650	96.57%	10,224,575
zygote		100	H3K27ac rep1	67,064,234	98.79%	23,260,997
		100	H3K27ac rep2	80,947,435	98.83%	16,875,059
	C57xPWK*	115	H3K27ac rep3	33,874,753	97.84%	15,002,433
			Input	67,538,080	97.99%	13,538,615
	C57xPWK*	172	H3K9me3 rep1	24,104,179	92.73%	2,121,116
	C57xPWK*	140	H3K9me3 rep2	34,558,216	62.49%	2,493,852
P300 KD zygote		50	H3K27ac rep1, P300 siRNA#1	30,084,333	98.21%	12,564,663
		50	H3K27ac rep2, P300 siRNA#2	38,051,184	97.13%	10,429,070
Late 2-cell		50	H3K27ac rep1	88,556,403	94.77%	6,076,030
		50	H3K27ac rep2	94,438,534	96.31%	3,985,965
	C57xPWK*	80	H3K27ac rep3	30,065,051	96.56%	11,210,820
			Input	81,723,616	97.59%	14,985,273
late 2-cell + amanitin		25	H3K27ac rep1	16,926,415	97.75%	10,551,390
		25	H3K27ac rep2	18,176,431	98.19%	12,178,819
late 2-cell + SAHA	C57xPWK*	65	H3K27ac rep1	57,973,288	97.21%	29,911,253
	C57xPWK*	50	H3K27ac rep2	32,369,420	96.98%	19,737,274
4-cell	C57xPWK*	25	H3K27ac rep1	29,218,773	95.72%	4,787,006
	C57xPWK*	25	H3K27ac rep2	30,757,112	94.44%	3,053,100
	C57xPWK*		Input	26,501,581	97.02%	7,066,625
4-cell + P300KD		41	H3K27ac rep1, P300 siRNA#1	35,056,763	94.48%	3,325,778
		40	H3K27ac rep2, P300 siRNA#2	35,572,091	75.98%	8,734,350
morula	C57xPWK*	10	H3K27ac rep1	42,902,648	95.13%	6,126,948
	C57xPWK*	10	H3K27ac rep2	28,558,500	95.38%	4,821,436
	C57xPWK*		Input	32,511,564	96.37%	7,085,509
blastocyst	C57xPWK*	5	H3K27ac rep1	79,510,593	94.66%	5,092,684
	C57xPWK*	5	H3K27ac rep2	86,512,908	94.01%	6,992,538
	C57xPWK*		Input	42,378,257	97.50%	13,360,793

* C57BL/6N females crossed with PWK/PhJ males.

Supplementary Table S3 Sequencing and mapping information of mouse H3K27ac ChIP-seq data.

Stage	Embryo Num.	Antibody	Raw reads	Mapping rate	Unique reads
Late 2-cell	50	H3K18ac rep1	29,666,908	97.15%	10,618,071
	50	H3K18ac rep2	29,181,812	96.67%	7,862,155
	50	H3K9ac rep1	37,717,696	96.34%	14,267,657
	50	H3K9ac rep2	37,169,159	96.83%	19,395,780
4-cell	25	H3K18ac rep1	29,427,252	96.43%	10,219,804
	25	H3K18ac rep2	28,235,340	96.04%	9,185,070
	25	H3K9ac rep1	25,575,953	94.94%	3,362,174
	25	H3K9ac rep2	26,535,498	75.77%	602,609
8-cell	25	H3K18ac rep1	31,252,682	95.73%	10,599,009
	25	H3K18ac rep2	32,766,271	96.43%	8,675,497
	25	H3K9ac rep1	33,210,477	95.68%	3,610,033
	25	H3K9ac rep2	36,131,048	95.53%	4,161,152
morula	10	H3K18ac rep1	29,855,773	97.07%	11,701,420
	10	H3K18ac rep2	30,415,943	95.34%	8,340,302
	10	H3K9ac rep1	56,567,715	77.61%	1,698,482
	10	H3K9ac rep2	49,237,990	93.26%	2,853,586
	10	H3K9ac rep3	18,465,717	86.73%	404,557
blastocyst	5	H3K18ac rep1	51,914,292	95.63%	13,010,020
	5	H3K18ac rep2	42,171,427	95.37%	10,570,992
	5	H3K9ac rep1	29,013,088	94.80%	2,943,047
	5	H3K9ac rep2	19,865,300	88.11%	717,245

Supplementary Table S4 Sequencing and mapping information of mouse H3K18ac and H3K9ac ChIP-seq data.

Supplementary Table S5 GO results of genes with different types of acetylations in promoters in mouse early embryos.
